# Genome-guided antimicrobial potential of *Bacillus stercoris* from coastal sand with activity against multidrug-resistant bacteria, including MRSA

**DOI:** 10.1007/s10123-026-00836-x

**Published:** 2026-05-01

**Authors:** Camilla Andrade Silva Valença, Bruno Andrade, Marcus Viana, Ana Andrea Teixeira Barbosa, Silvio Santana Dolabella, Patrícia Severino, Guillaume Ménard, Yoann Augagneur, Bertram Brenig, Eliana B. Souto, Vasco Azevedo, Eric Guedon, Sona Jain

**Affiliations:** 1 Postgraduate Program in Industrial Biotechnology, Tiradentes University, Aracaju, Sergipe Brazil; 2 State University of Southwest Bahia, Jequié, Brazil; 3 Federal University of Minas Gerais, Belo Horizonte, Brazil; 4 Postgraduate Program in Biology of Infectious and Parasitic Agents, Federal University of Sergipe, São Cristóvão, Sergipe, Brazil; 5 Bacterial Regulatory RNAs and Medicine (BRM) Rennes, Univ de Rennes, INSERM, Rennes, France; 6 Georg August University Göttingen, Göttingen, Germany; 7 UCD School of Chemical and Bioprocess Engineering, University College Dublin, Belfield, Dublin 4, D04 V1W8 Ireland; 8 STLO, INRAE, L’Institut Agro, Rennes, France; 9 Postgraduate Program in Biotechnology (RENORBIO), Federal University of Sergipe, São Cristóvão, Sergipe Brazil

**Keywords:** Antimicrobial activity, Methicillin-resistant *S*. *aureus*, Methicillin-susceptible *S. aureus*, Secondary metabolites, *In vitro* analysis, *In silico* analysis

## Abstract

Coastal beach environments harbor diverse bacterial communities, yet their antimicrobial potential remains largely unexplored. This study evaluates the antimicrobial potential of a bacterial isolate 2AT10 from Brazilian beach sand through *in vitro*, *in silico*, and *ex vivo* assays. Isolate 2AT10, a rod-shaped Gram-positive bacterium, exhibited broad-spectrum antimicrobial activity, with its culture supernatants showing greater efficacy against Gram-positive bacteria, including multidrug-resistant strains such as methicillin-resistant *Staphylococcus aureus* (MRSA). Antimicrobial activity from two differently processed supernatants decreased after heat treatment, enzymatic digestion, and pH changes, suggesting similar proteolytic profiles. Fourier-transform infrared spectroscopy (FTIR) revealed peaks corresponding to C = O and NO₂ groups, indicating the presence of amide functional groups. Importantly, the supernatants did not cause irritation in the *ex* *vivo* chorioallantoic membrane assay. Genomic analysis identified isolate 2AT10 as *Bacillus stercoris*, harboring genes encoding subtilosin A, bacilysin, bacillibactin, bacillaene and lactococcin-like bacteriocins, all associated with antimicrobial activity. A mutation in the sboA gene, involved in subtilosin A biosynthesis and previously linked to *in vitro* hemolytic activity and enhanced activity against Gram-positive bacteria, was also detected. Molecular docking and dynamics analyses suggested a possible interaction between lactococcin-like bacteriocin and a membrane component of the *S. aureus* phosphotransferase system transporter, possibly increasing membrane permeability.

**Graphical Abstract:**

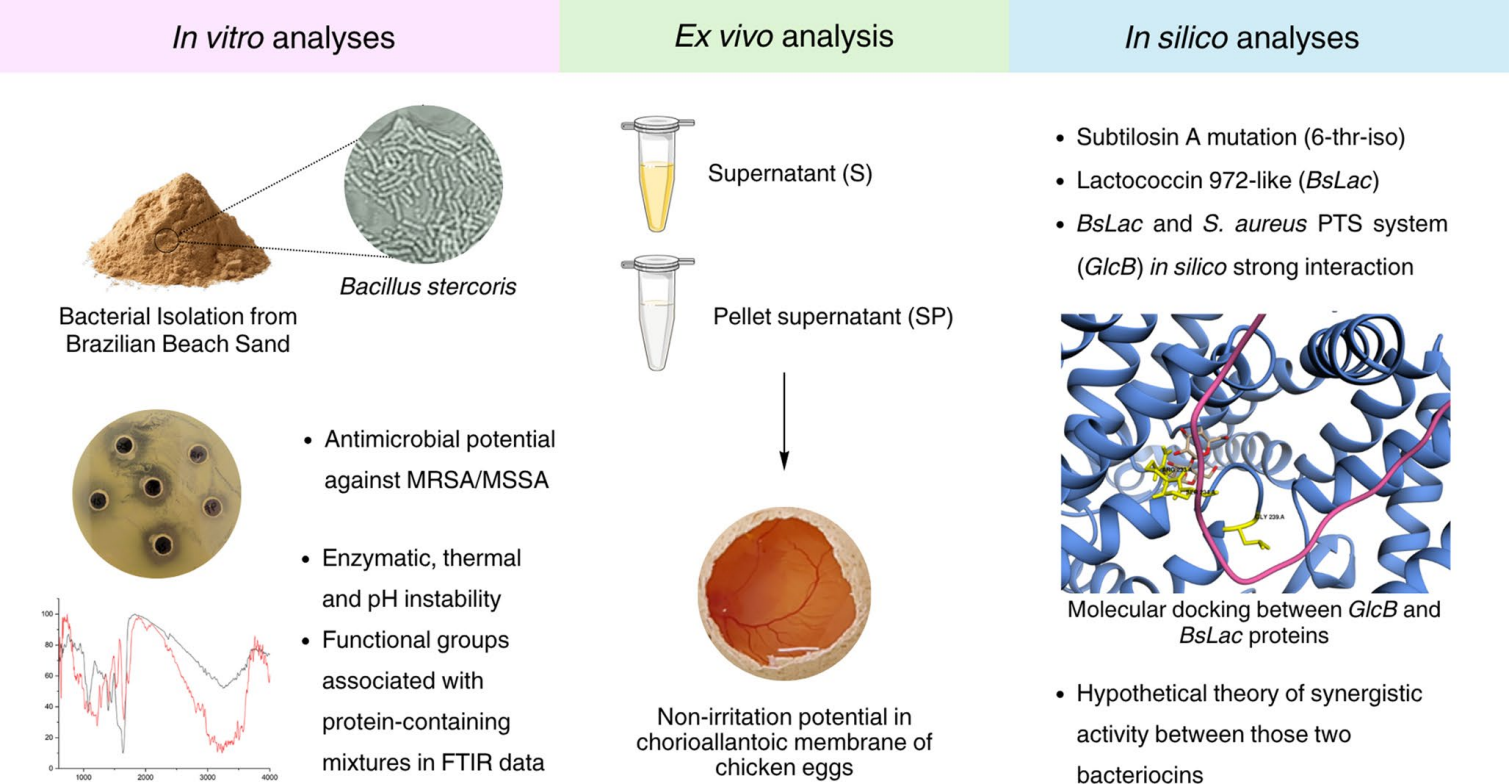

**Supplementary Information:**

The online version contains supplementary material available at 10.1007/s10123-026-00836-x.

## Highlights

Genomic analysis of *Bacillus stercoris* isolated from beach sand.

Bacterial supernatant with strong activity against Gram-positive bacteria including multidrug-resistant strains.

Surfactin, bacillaene, fengycins, bacillibactin, bacilisin, zwittermicin A and lactococcin 972-like bacteriocin predicted in the bacterial genome.

First preliminary *in silico* insights of interaction between lactococcin 972-like bacteriocin and membrane-bound *S. aureus* glucose transporter.

**Supplementary Information:**

The online version contains supplementary material available at 10.1007/s10123-026-00836-x.

## Background

With the rising resistance to conventional antimicrobials, research in recent decades has primarily focused on discovering new antimicrobial compounds effective against resistant microorganisms. Bacterial metabolites, such as bacteriocins, lipopeptides, siderophores, and volatile compounds, have been widely studied. Bacteriocins, defined as ribosomally synthesized antimicrobial peptides, include compounds, such as lactococcin, produced by *Lactococcus* spp., and subtilosin, produced by *Bacillus* spp (Şahingil et al. 2011, Dinesen et al. 2025, Zhao et al. 2022). Lactococcin and subtilosin have been associated with membrane damage and significant activity against Gram-negative and Gram-positive bacteria (Martinez et al. 2000, Algburi et al. 2017, Öncül and Yıldırım 2019). Class IIa and IId bacteriocins, including the Lactococcin 972, are non-lantibiotic and non-pediocin-like bacteriocins which can act directly on the phosphotransferase permease carbohydrate transport systems (PTS) (Martinez et al. 2000, Hernández-González et al. 2021, Martinez et al. 1999).

Coastal beaches are popular leisure locations of millions of people worldwide and are home to many microbial species, mainly in the sand. Most studies involving this type of sample focus on the potential danger to human health represented by the pathogenic microbial populations associated with sand (Whitman et al. 2016, Teixeira et al. 2020, Degenhardt et al. 2020). A recent study aiming to characterize the pathogenic potential of beach sand microbiomes reported that around 19.75% of bacteria isolated from this environment belonged to the genus *Bacillus*, highlighting the prevalence of species such as *Bacillus halotolerans*,* Bacillus circulans*,* Bacillus cereus*, and *Bacillus thuringiensis* (Soffritti et al. 2023). Based on comparative genomic analyses *Bacillus subtilis* subsp. *stercoris* was recently reclassified as a distinct species, i.e., *Bacillus stercoris*. Mostly isolated from soil, this species is associated with plant and animal waste residues (Singh et al. 2024, Chouaia and Dittmer 2024). *B. stercoris* has been reported to produce several secondary metabolites that differ from those of other related species (Dunlap et al. 2020). Most articles have discussed the activity of this species against fungal plant pathogens, such as *Colletotrichum gloeosporioides*, *Coniothyrium diplodiella*, *Fusarium* spp., and *Botrytis cinerea*, as well as its role in promoting plant growth (Wang et al. 2021, Pengproh et al. 2023).

Although *Bacillus* spp. is known to possess antimicrobial activity and to produce compounds, such as bacteriocins, little attention has been paid to *B. stercoris*. For the first time, we propose a correlation between the genomics of *B. stercoris* and its *in vitro* activity against various antibiotic-resistant bacterial pathogens, including methicillin-resistant *Staphylococcus aureus* (MRSA). Additionally, for the first time we present *in silico* insights of an interaction between lactococcin 972- like bacteriocin (predicted in the bacterial genome) and the *S. aureus* phosphotransferase system transporter.

## Methods

### Sample Collection and Isolation

Sand sample from Atalaia Beach (10.997482, -37.052538), located in Aracaju-SE (Brazil), was used in this study. The sample was collected from three equidistant points, with 3 cm depth of dry and wet sand, using sterile Falcon tubes, which were then refrigerated until processing (Prashanthi et al. 2021). First, 2 g of the sand sample was homogenized with 10 mL of saline phosphate buffer. The contents were then centrifuged at 12,000 × g for 10 min at 20 °C, and 100 µL of the obtained supernatants (non-diluted and 10^− 2^ and 10^− 4^ diluted) were plated on nutrient agar (KASVI^®^) medium and incubated at 37 °C for 24 h (Khaleghi et al. 2019). Bacterial colonies with distinct morphology were tested for their antimicrobial property.

## Bacterial strains

The ATCC indicators used in this study include *Escherichia coli* (ATCC 23226 and ATCC 25922), *Staphylococcus aureus* (ATCC 8095 and ATCC 25923), *Enterococcus durans* (ATCC 551225), *Pseudomonas aeruginosa* (ATCC 27853 and ATCC 27853), *Klebsiella pneumoniae* (ATCC 13883), and *Acinetobacter baumannii* (ATCC 19606).

Laboratoire de Biochimie Pharmaceutique—Bacterial RNAs & Medicine (BRM—Université de Rennes) provided a panel of Gram-positive and Gram-negative bacteria, including multidrug-resistant (MDR) bacteria. Specific details are listed in Table S1.

## Antimicrobial Screening

 The agar overlay methodology was used for the first screening (Carvalho et al. 2006). As described before, nine ATCC indicator strains were used to screen antimicrobial activity. All the indicator strains were grown in Nutrient Broth (NB, KASVI^®^) at 37 °C overnight, 150 rpm, and used at 1 × 10^8^ CFU/mL (colony-forming unit) concentration.

## Morphological Characterization and Antibiogram

Isolate 2AT10 was analyzed for its growth characteristics in nutrient agar and blood agar, as well as via Gram staining, by optical microscopy (Olympus^®^ CX23) and contrast phase microscopy (Olympus^®^ CKX53). The antibiogram was performed according to the Clinical and Laboratory Standards Institute (CLSI).

## Antimicrobial activity of the Supernatants S and SP

Isolate 2AT10 was cultured in NB at 37 °C under static conditions for various incubation periods (12, 24, 48, and 72 h). Its antimicrobial activity against *S. aureus* ATCC 25,923 was initially assessed using two types of supernatants (S and SP), following the method described by de Carvalho et al. (2006). This strain was selected as the indicator due to its higher susceptibility in the previous experiments. The supernatant S was obtained after centrifugation of the bacterial culture at 12,000 × g for 25 min at 4 °C. The bacterial pellet obtained after centrifugation was resuspended in 0.9% saline solution (pH 2), incubated for 1 h at room temperature with vortex stirring every 15 min and then centrifuged again (12,000 × *g* for 25 min at 4 °C). The supernatant collected from this step constituted the SP fraction. S and SP were also evaluated for their thermal (40°, 50°, 60°, 70°, 80°, 90 °C for 20 min and 100 °C for 15 min), proteolytic (treatment with 1 mg/mL proteinase K for 1 and 2 h at 37 °C) and pH stability (2, 5, 7, and 10) (Anvisa 2008, Reda and Refaie 2019). Antimicrobial activity was tested for these evaluations against both *S. aureus* ATCC 25923 and *P. aeruginosa* ATCC 27853. Antimicrobial activity of S and SP exposed to these various treatments was compared to that of untreated S and SP. 

### Bacterial Growth and Activity Against Methicillin-Resistant *S. aureus*

The growth of 2AT10 was monitored by determining the optical density (OD) at 600 nm (OD600) during 72 h, at 37^°^C under static conditions in Luria-Bertani (LB), NB, and Modified Medium (MOD) prepared as described by Jamil et al. (2007). As described above, 25 µL of 0.5 OD starter culture was used in 3 mL of culture media for growth curve analysis. To evaluate the antimicrobial activity of the isolate 2AT10 against methicillin-resistant *S. aureus* N315, supernatant S was collected at 24, 48, and 72 h from bacterial cultures in LB, NB, and MOD. The agar well diffusion method described by Amiri et al. (2022) was used to analyze the antimicrobial activity. Later, other antibiotic-resistant clinical bacterial isolates, as described before, were also used as indicators for assessing antimicrobial susceptibility. For this, the isolate 2AT10 was grown in MOD, 24 h, 37 °C, under static conditions, and supernatant S was used for the antimicrobial assay.

### Fourier Transform Infra-Red (FTIR) Analysis of the Supernatants

 Fourier Transform Infra-Red (FTIR) analysis was performed using a spectroscope coupled with a diamond/zinc selenide (ZnSe) crystal and an attenuated total reflection (ATR) device (IRAffinity-1 S, Shimadzu, Kyoto, Japan) in the ZnSe spectral range of 400–4000 cm^− 1^ with a resolution of 4 cm^− 1^ and 128 scans. The measurements obtained were treated using Labsolutions software (Yu et al. 2021).

## Hen’s Egg Test-Chorioallantoic Membrane (HET-CAM)

To qualitatively evaluate the irritation potential of the supernatants S and SP, the *ex vivo* Hen’s Egg Test Chorioallantoic Membrane (HET-CAM) test was performed according to the protocols (Luepke 1985, Palmeira-de-Oliveira et al. 2018, Machado et al. 2023) using fertilized chicken eggs (Asa Branca - São Cristóvão). NaOH (1 M) and NaCl (0.9%) solutions were used for positive and negative controls, respectively. 

## Genome Sequencing, Annotation, and Assembly

Isolate 2AT10 was cultured in NB at 37 °C, 12.000xg, overnight, and the cell pellet obtained after centrifugation was used for genomic DNA extraction. DNA was extracted using the Wizard^®^ Genomic DNA Purification Kit (Promega Corporation, USA), following the manufacturer’s instructions. The whole genome sequencing was performed using the Illumina^®^ HiSeq 2500 platform (California, USA), and 150 bp paired-end libraries were constructed with the ThruPLEX DNA-Seq Kit (Takara). Quality analyses and trimming of the sequencing reads were performed with the programs FastQC v. 0.12.1 (https://github.com/s-andrews/FastQC*)* (Wingett and Andrews 2018) and Fastp v. 0.23.3 (https://github.com/OpenGene/fastp*)* (Chen et al. 2018) respectively. The genome was assembled using Unicycler v. 0.5.0 (https://github.com/rrwick/Unicycler*)* (Wick et al. 2017). CheckM2 v. 1.0.2 (https://github.com/chklovski/CheckM2*)* (Chklovski et al. 2023) was used to assess the percentage of completeness and contamination, and GUNC v. 1.0.6 (https://github.com/grp-bork/gunc*)* (Orakov et al. 2021) was used to identify contaminating contigs. The assembly fragmentation was evaluated using the QUAST v. 5.0.2 program (https://github.com/ablab/quast*)* (Gurevich et al. 2013). MOB-suite v. 3.1.9 (https://github.com/phac-nml/mob-suite*)* (Robertson and Nash 2018) was used to identify, assemble, and type plasmids and other mobile genetic elements. The genome was deposited in GenBank, which was annotated using the NCBI PGAP (https://github.com/ncbi/pgap*)* (Gen Bank ID GCA_041765565.1, RefSeq ID GCF_041765565.1) (Tatusova et al. 2016).

### Taxonomic Identification and Phylogenomics

The 2AT10 and related genomes were subjected to two different analyses for taxonomic identification. First, the Type (Strain) Genome Server – TYGS (https://tygs.dsmz.de/*)* (Meier-Kolthoff and Göker 2019), which uses digital DNA-DNA Hybridization (dDDH), was used to compare the query genome to a database of type strains genomes, and a dDDH cutoff value above 70% for genomes from the same species was considered. In the second step, the Genome Taxonomy Database Toolkit (GTDB-Tk) (https://github.com/Ecogenomics/GTDBTk*)* (Chaumeil et al. 2019), which uses the Average Nucleotide Identity (ANI) of a query genome against the Genome Taxonomy Database (GTDB) (https://gtdb.ecogenomic.org/), was used to identify the genome species. The ANI value within genomes from the same species is above 95% (Rodriguez et al. 2024).

*B. stercoris* was initially classified as a subspecies of *B. subtilis* (Dunlap et al. 2020). To support the reclassification as a separate species, we compared *B. stercoris* genomes to *B. subtilis* and five other closely related species using ANI and a phylogenetic tree. Besides 2AT10, 16 other *B. stercoris* genomes were downloaded from GenBank (July 2024) and checked for taxonomy and contamination. The ANI analysis was performed using pyANI v. 0.2.12 (https://github.com/widdowquinn/pyani*).* The phylogenetic tree was built using OrthoFinder v. 2.5.5 (https://github.com/davidemms/OrthoFinder) with the parameters “-M msa -T fasttree,” which implies using MAFF v7.525 for multiple sequence alignment and FastTree v. 2.1.11 for phylogenetic inference. *Bacillus amyloliquefaciens* DSM7 (GCF_000196735.1) was used as the outgroup, and the tree was visualized using iTOL (https://itol.embl.de/*)* (Table S2).

### Secondary Metabolites Prediction

Secondary metabolite predictions were initially generated by antiSMASH 7.0 (https://antismash.secondarymetabolites.org/*)* (Medema et al. 2011). In addition, the genome-wide functional annotation tool eggNOG mapper (http://eggnog-mapper.embl.de/*)* (Cantalapiedra et al. 2021) was used to identify metabolic pathways potentially involved in producing secondary metabolites. UniProt (https://www.uniprot.org/*)* and Blastp (blast.ncbi.nlm.nih.gov) were used to confirm the eggNOG mapper predictions. Furthermore, bacteriocin and RiPP genes were identified using the BAGEL 4 tool (http://bagel4.molgenrug.nl/*)* (de Jong et al. 2006). To evaluate the mutations in the *sbo-alb* locus genes, the amino acid sequences relative to the identified respective genes in the 2AT10 genome were compared to all related genes found in the UNIPROT database (https://www.uniprot.org*)* using Jalview 2.11.4.1 (https://www.jalview.org/*).*

### Computational bioactivity prediction

The structure of one member of lactococcin-family identified in the *B. stercoris* genome (BsLac) was modeled and validated using the Alphafold 3 server (https://alphafoldserver.com/) (Abramson et al. 2024). In addition, the 3D structure of BsLac was subjected to 5,000 cycles of conjugate gradient energy minimization, followed by two isobaric (1 ATM) and isothermal (310 K) molecular dynamics (MD) equilibration steps using GROMACS 2025 (Spoel et al. 2005) for 1 nanosecond (ns) each. Furthermore, a stability MD simulation of 5 ns under the same conditions was performed to resolve any clashes in the protein structure.

To investigate the potential mechanism of action of BsLac against *S. aureus*, we analyzed the 3D structure of GlcB (UniProt ID: Q7A3G4) to assess BsLac’s ability to block the transmembrane TM7 region, which is responsible for glucose translocation into the bacterial cytoplasm (McCoy et al. 2016). Additionally, we performed a BLASTp search using the amino acid sequence of BsLac from *B. stercoris* against the UniProt database to evaluate its similarity to class IId lactococcins. Homologous sequences were further aligned using the Clustal Omega server (Sievers and Higgins 2018).

Proteins were prepared for molecular docking by defining the GlcB TM7 region amino acids. For this, the template 5IWS from *B. cereus* BcMalT Maltose Transporter (McCoy et al. 2016) was used, as it shares homology with GlcB, to perform a structural alignment using ChimeraX (Pettersen et al. 2004), where the conserved amino acid His240 from 5IWS corresponds to Gly239 in the GlcB structure, as well as Ser234 and Leu240. Thus, this region was defined as the GlcB TM7 region for docking with BsLac. Docking calculations were carried out using the HADDOCK server (Honorato et al. 2024), targeting the TM7 region of GlcB and utilizing the complete BsLac structure with default parameters. The best complexes were selected based on the most negative docking scores and the positioning of the BsLac structure within the GlcB TM7 pocket.

The best GlcB-BsLac complex was subjected to a 500 ns stability MD simulation using GROMACS 2025. Using the CHARMM-GUI server (Park et al. 2023), the complex was prepared for this. The proteins were subjected to 5.000 cycles of conjugate gradient energy minimization, followed by an equilibrium dynamics step at constant volume and temperature (NVT) for 1 ns and a second equilibrium step at continuous pressure and temperature (NPT), both using the Berendsen thermostat (Lemkul 2018). Furthermore, we proceeded with a 500 ns stability MD simulation using the same temperature and pressure parameters described above. The stability of the complex was measured by the overall backbone RMSD of the GlcB TM7 region and the fluctuations of the amino acids in the same region. In addition, the number of hydrogen bonds formed during the MD simulation and the system’s energy were evaluated.

### Statistical Analysis

All experiments were performed in triplicate and repeated in at least two independent experiments. Results are presented as the mean ± standard deviation of biological replicates. For absorbance measurements, values were normalized to the control condition. Statistical differences between groups were analyzed using two-way (2-way) ANOVA, For the analysis of bacterial growth and antimicrobial activity, incubation time and supernatant type were compared, while for statistical analyses of growth (OD), time and culture medium were compared. For heat, pH, and enzyme treatments, treated and control groups were compared. When significant effects were detected, multiple comparisons were performed using Tukey’s post hoc test to determine pairwise differences between groups. Statistical significance was considered at *p* < 0.05. All analyses were conducted with GraphPad Prism (GraphPad Software, San Diego, CA, USA). 

## Results

### Bacterial Isolate and Antimicrobial Potential

A total of eight morphologically distinct colonies were obtained, of which only isolate 2AT10 was selected for further study based on its superior antimicrobial activity. Isolate 2AT10 inhibited the growth of ATCC indicator strains, such as *S. aureus*, *S. epidermidis*,* P. aeruginosa*, and *E. coli* (Fig. S1). No significant inhibition was recorded against *E. durans*, *K. pneumoniae*, and *A. baumannii* (Table 1).

**Table 1:** Inhibition profile of the isolate 2AT10 from Atalaia Beach, using the agar overlay method

Pathogenic Strains	Inhibition Zones (mm)
*Staphylococcus aureus* ATCC 8095	35 ± 0.2
*Staphylococcus aureus* ATCC 25923	40 ± 0.1
*Staphylococcus epidermidis* ATCC 12228	20 ± 0.1
*Pseudomonas aeruginosa* ATCC 27853	27 ± 0.3
*Pseudomonas aeruginosa* ATCC 27583	25 ± 0.1
*Escherichia coli* ATCC 23226	No activity
*Escherichia coli* ATCC 25922	35 ± 0.1
*Enterococcus durans* ATCC 551225	No activity
*Klebsiella pneumoniae* ATCC 13883	No activity
*Acinetobacter baumannii* ATCC 19606	No activity

**Fig. 1:**
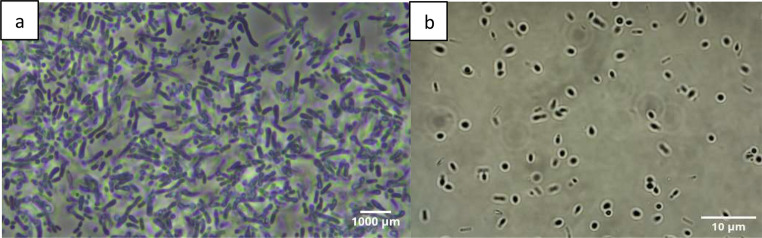
Microscopy images **(a)** optical at 40x of 2AT10 vegetative cells, and **(b)** contrast-phase at 100x of the spores

Gram staining and light microscopy analysis of 2AT10 identified it as a sporulating, rod-shaped, Gram-positive bacterium (Fig. 1A, B). On solid media, 2AT10 grew as dry and whitish colonies with irregular edges **(**Fig. S2), and in liquid medium, biofilm formation was shown during static growth conditions. On blood agar, 2AT10 was found to be alpha-hemolytic. Furthermore, 2AT10 was sensitive to all the tested antibiotics (Table S3).

### Antimicrobial Activity of 2AT10-derived Supernatants

Supernatants S and SP displayed antimicrobial activity against *S. aureus* ATCC 25923 after testing different incubation times (Table 2)**.** An incubation time of 48 h was selected for all the subsequent analyses.

**Table 2:** Measurement of inhibition zones diameters of the 2AT10-derived supernatants S and SP against *S. aureus* ATCC 25923 using agar diffusion method. Supernatants were obtained from 2AT10 cultures at different incubation periods (12, 24, 48, and 72 h) under static conditions in NB

Time (h)	Inhibition zones (mm) against *S. aureus* ATCC 25923	
	Supernatant S	Supernatant SP
12	8.0 ± 0.1	10.6 ± 0.2
24	8.4 ± 0.1	12.6 ± 0.3
48	10.1 ± 0.1	10.7 ± 0.2
72	10.0 ± 0.2	12.6 ± 0.5

* Antimicrobial activity of S and SP (2-way ANOVA, *p* = 0.00135) was statistically different and was dependent on incubation times (2-way ANOVA, *p* < 0.0001)

### Enzymatic, Thermal, and pH Stability of 2AT10-derived Supernatants

**Fig. 2:**
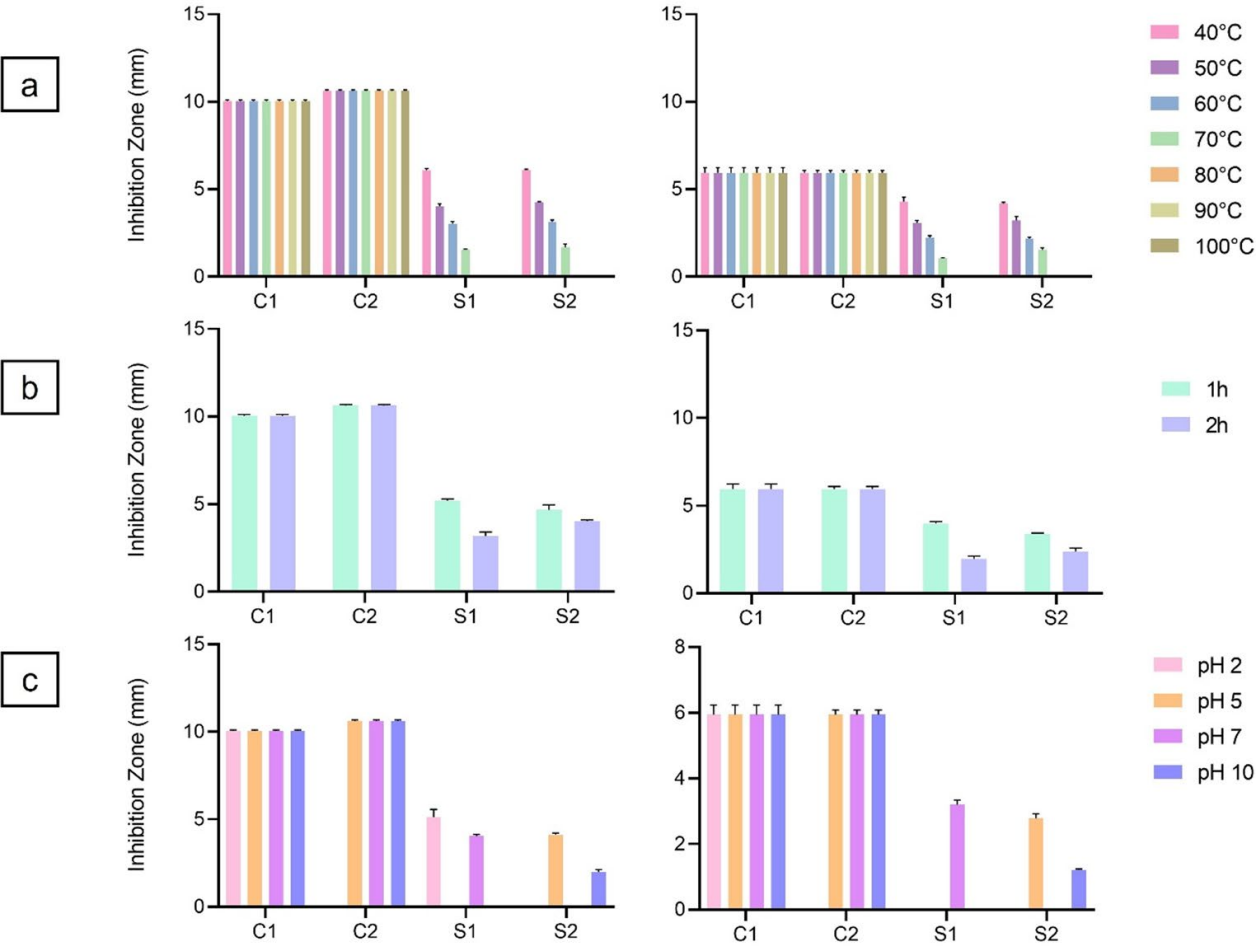
Graphs related to antimicrobial activity of 2AT10-derived supernatants against *S. aureus* ATCC 25923 (left panel) and *P. aeruginosa* ATCC 27853 (right panel), after **(a)** thermic, **(b)** enzymatic, and **(c)** pH treatments, where C1 is the untreated supernatant S; C2 - is the untreated pellet supernatant SP; S1- treated supernatant S; S2- treated pellet supernatant SP. Antimicrobial activities of controls and treatments were statistically different (2-way ANOVA, *p* < 0.05). Absence of bars represents complete loss of activity after treatment

One Gram-positive (*S. aureus* ATCC 25923) and one Gram-negative (*P. aeruginosa* ATCC 27853) indicator strain that gave positive results in initial antimicrobial tests were utilized for this analysis. Upon treatment with proteinase K, the antimicrobial activity associated with S and SP fractions decreased against *S. aureus* ATCC 25923 and *P. aeruginosa* ATCC 27853 (Fig. 2; Table S4). Also, the loss of activity was progressive with the enzyme treatment time. The results suggest that the two supernatants may have similar proteolytic metabolic profiles. It is worth mentioning here that SP (supernatant from pellet) probably contains only the molecules that remain attached to the bacterial membrane and separate in the 0.9% saline (pH 2) during the 1-hour agitation (Todorov 2009).

As shown in Fig. 2, thermal treatment decreased the antimicrobial activity of both S and SP against the two indicator strains. The temperature impact was limited at lower ranges, with activity decreasing to 26–42% at 40 °C and 43–60% at 50 °C. When exposed to 60° and 70 °C, only 15–35% of the initial inhibitory activity of S and SP was maintained, while exposure to 80° and 100 °C led to a complete loss of their antimicrobial activity. Supernatant S showed greater thermal sensitivity with a reduction in activity starting at 40 °C, while for SP, this reduction was observed only starting at 60 °C. Both supernatants were more stable from an enzymatic standpoint than thermal and pH conditions. Decreased antimicrobial profiles were observed for both S and SP at all pH conditions tested.

### Antimicrobial Potential against MRSA

The influence of the culture medium on growth and the antimicrobial activity 2AT10 was also assessed against the MRSA strain N315. For this, 2AT10 was cultivated in three different media (LB, NB, and MOD), and the supernatant S was used for the antimicrobial assays **(**Table 3; Fig. S3). Optical density in LB was higher than in NB and MOD, but the biomass increase did not promote the antimicrobial activity. Overall, the bacterial growth in MOD displayed the greatest antimicrobial activity when compared to LB and NB, which were statistically different (2-way, ANOVA, *p* < 0,0001). These results suggest that the *in vitro* antimicrobial activity observed is not strictly related to the quantity of cells.

**Table 3:** Optical density (OD) at 600 nm and inhibition zone (IZ) diameters (in mm) measured for the supernatant S collected from bacterial cultures grown in Luria-Bertani Broth (LB), Nutrient Broth (NB), and Modified Medium (MOD) after 24, 48, and 72 h of incubation against methicillin-resistant *S. aureus* N315, using well diffusion method

Culture medium	Optical Densities (OD at 600 nm) and Inhibition Zones (IZ in mm)					
	24 h		48 h		72 h	
OD	IZ	OD	IZ	OD	IZ	
LB	1.50*	19.5 ± 0.3	1.29*	23.10 ± 0.20	1.07*	24.6 ± 0.3
NB	0.99*	19.6 ± 0.3	0.69*	21.65 ± 0.15	1.19*	19.0 ± 0.1
MOD	0.53*	32.5 ± 0.2	0.60*	19.7 ± 0.19	0.66*	29.6 ± 0.4

*No standard deviation was observed between replicates when measuring OD values

Table 4 summarizes the antimicrobial activity of 2AT10 against various clinically relevant, antibiotic-resistant pathogenic strains. Growth inhibition was observed in both methicillin-resistant and vancomycin-resistant clinical isolates. Notably, *S. aureus* P333 (LV CA MRSA), associated with skin infections transmitted by close contact, exhibited one of the most significant inhibition zones, followed by other MRSA clinical isolates (Sassi et al. 2017). Results were similar to those of MDR *Staphylococcus haemolyticus* isolates. Conversely, no activity was observed with vancomycin-resistant *Enterococci* and Gram-negative bacteria. These results support the association between the bacterial supernatants and a more potent inhibitory effect, mainly against *Staphylococcus* sp., independently of their antibiotic-resistant status.

**Table 4:** Bacterial inhibition zones of various strains treated with 2AT10-derived supernatant S using the well diffusion method. Legend: ST (Sequence Type), MSSA (methicillin-susceptible *S*. *aureus*), MRSA (methicillin-resistant *S*. *aureus*), VRSA (vancomycin-resistant *S*. *aureus*), GISA (glycopeptide-intermediate *S*. a*ureus*), meti (methicillin), vanco (vancomycin), VRE (vancomycin-resistant *Enterococci*), ESBL (extended-spectrum beta-lactamase), CPE (carbapenem-producing *Enterobacterales*)

Species	Resistance indicators	ST	Strains	Inhibition zone (mm)
*Staphylococcus aureus*	MSSA	7	IOA14	20 ± 0
		8	UAMS1	19.5 ± 0.7
			Newman	20 ± 0
			S20	24 ± 0
			Thom	23 ± 0.1
		25	P19	25 ± 0
		45	P8	25.5 ± 0.7
		188	C57	23 ± 0
	MRSA	8	USA300 LAC	21 ± 0
			P333	24.5 ± 0.7
			S43	20 ± 0
			C26	22.5 ± 0.7
		88	CA1	15.5 ± 0.7
		398	LA1	23 ± 0
		398	LA2	24.5 ± 0.7
	MSSA/VRSA	72	Van 035	24.5 ± 0.7
		239	van 023	Partial inhibition
	MRSA/GISA	5	Mu50	22.5 ± 0.7
*Staphylococcus haemolyticus*	Meti-R, vanco-S	29	STHA1	19 ± 0.1
	meti-R, vanco-R		STHA2	17 ± 0.7
*Enterococcus faecium*	VRE	17	Aus 004	No activity
*Klebsiella pneumoniae*	ESBL	ND	KLPN1	No activity
*Enterobacter cloacae*			ATCC 13047	No activity
Complex	ESBL + NDM1	111	ECC1	No activity
*Citrobacter freundii*	ESBL + NDM1	408	CIFR1	No activity

### FTIR Profile of the Supernatants

Although both supernatants exhibited similar overall FTIR profiles, subtle differences in spectral features were observed. In the 2300–3700 cm⁻¹ region, SP showed lower transmittance values than S, suggesting differences in the overall composition or concentration of chemical constituents between the two crude mixtures (Fig. S4). A band near 1531 cm⁻¹ was observed in SP but not in S; while this region is often associated with N–O stretching vibrations, its assignment in a complex mixture remains tentative. In supernatant S, a broad band around 3250 cm⁻¹ is consistent with O–H stretching, typically associated with hydroxyl-containing compounds or water. A band near 1637 cm⁻¹, present in both samples, is characteristic of C = O stretching or N–H bending modes commonly found in protein-containing mixtures. Additionally, SP displayed lower transmittance in regions above 2000 cm⁻¹, further supporting differences in composition between the two supernatants. However, as these samples represent unfractionated mixtures, the spectra reflect overlapping signals from multiple components. Therefore, these features should be interpreted as general chemical fingerprints rather than evidence for specific metabolite classes.

### Non-Irritative Effect of 2AT10-derived Supernatants

**Fig. 3:**
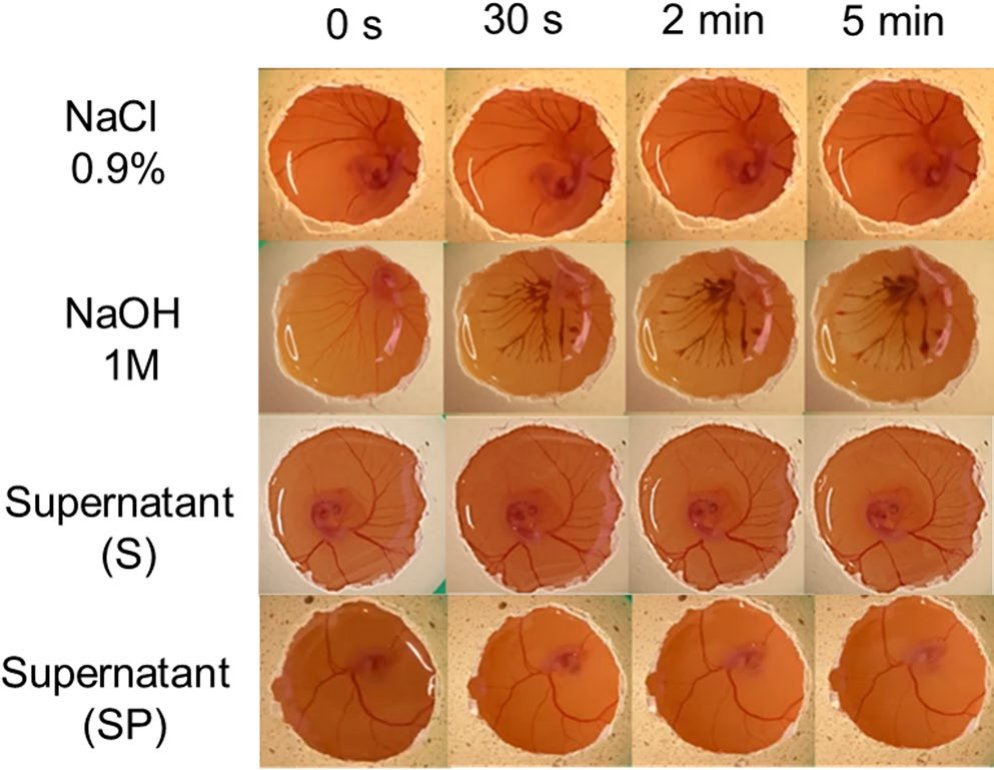
Hen’s egg chorioallantoic membranes at times 0 s, 30 s, 2 min, and 5 min exposed with 2AT10-derived supernatants S and SP and the respective negative (NaCl 0.9%) and positive (NaOH 1 M) controls

S and SP showed no modifications to the egg membrane, with a total absence of irritation. The membranes treated with the negative control also showed no modifications as expected, while the positive control generated intense hemorrhage within the first 30 s of exposure (Fig. 3).

### Taxonomic Identification and Phylogenomics

The 2AT10 genome (Gen Bank ID GCA_041765565.1, RefSeq ID GCF_041765565.1, Acession Number JBEMBP00000000) was classified as *B. stercoris* by both TYGS and GTDB-TK, with DDH and ANI values above 70 and 95%, respectively, in comparison to the type strain. The genome was assembled as a draft genome with 40 contigs, 4,108,661 bp, 43.74% GC content, and no plasmid. The genome annotation identified 4,156 CDS genes, 70 tRNA genes, 5 rRNA genes (1 complete, 4 partial), and 106 pseudogenes. Minimum information about the genome sequence (MIGS) of *B. stercoris* 2AT10 is presented in Table S5.

**Fig. 4:**
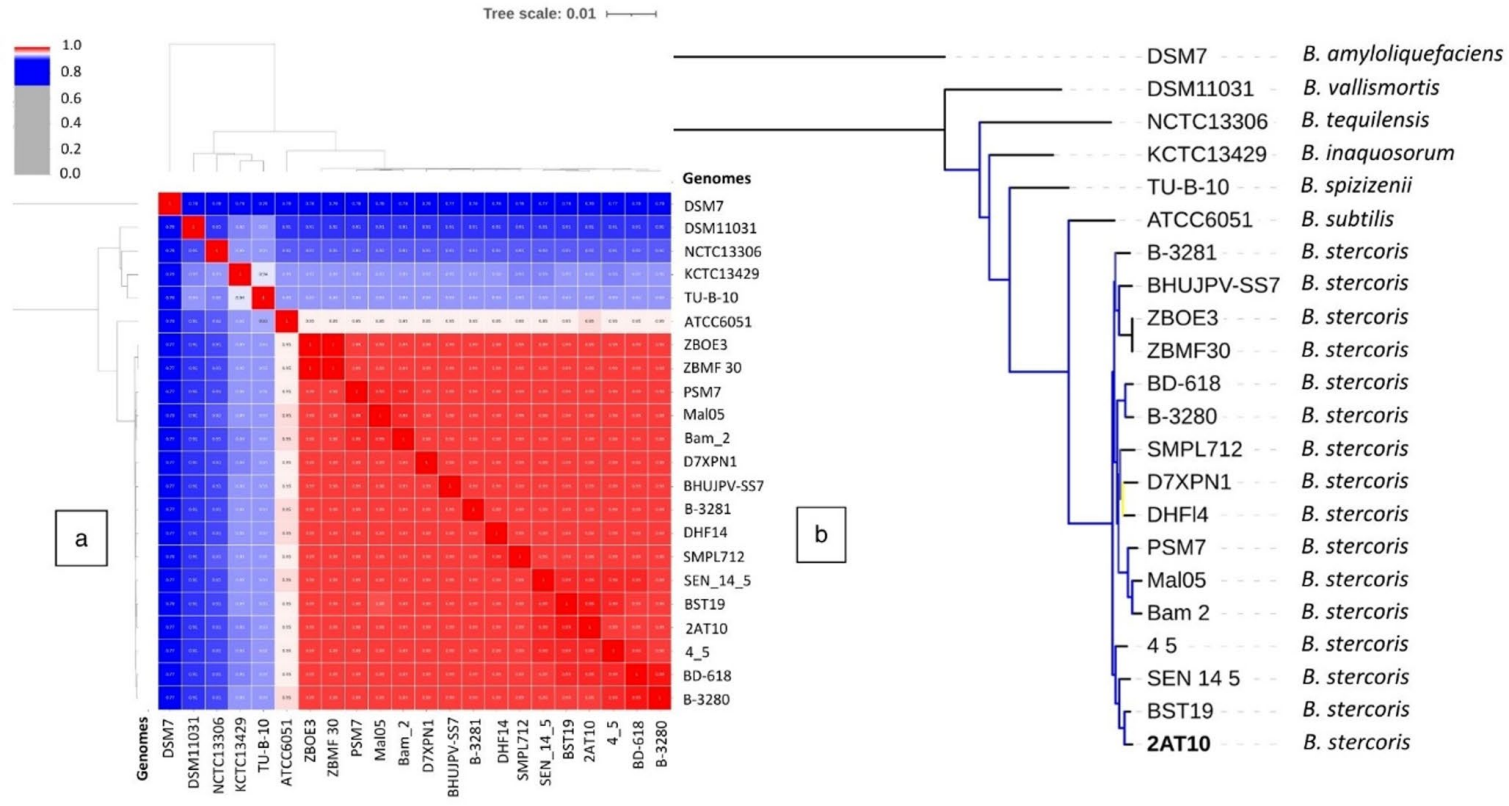
**(a)** ANI heatmap showing the values within *Bacillus stercoris* and the type strains of other *Bacillus* species: *Bacillus amyloliquefaciens* DSM7, *Bacillus inaquosorum* KCTC 13429, *Bacillus spizizenii* TU-B-10, *Bacillus subtilis* ATCC 6051, *Bacillus tequilensis* NCTC 13306, *Bacillus vallismortis* DSM 11031, as external outgroups, and **(b)** Phylogenetic tree of *B. stercoris* and other *Bacillus* species. The tree was built using OrthoFinder v. 2.5.5 pipeline with the parameters “-M msa -T fasttree”, from the alignment of 1,254 single-copy genes

All 16 public *B. stercoris* genomes analyzed in this study were classified as *B. stercoris*. The ANI values ranged from 95.18 to 95.35% between *B. subtilis* type strain and *B. stercoris* genomes, while within *B. stercoris* genomes, the values ranged from 98.5 to 99.99%. The phylogeny built from the alignment of 1.254 shared proteins formed a single clade with all the *B. stercoris* strains, with *B. subtilis* as a sister clade (Fig. 4).

#### Genes Coding for Secondary Metabolites Production in the 2AT10 Genome

Analyses using antiSMASH 7.0 and BAGEL 4.0 identified 12 biosynthetic gene clusters (BGCs) associated with secondary metabolite production. Among these, nonribosomal peptide synthetases (NRPS) and type III polyketide synthases (T3PKS) were the most prevalent (Table 5). The sactipeptide cluster (from the RiPP class, standing for ribosomally synthesized and post-translationally modified Peptides) also yielded 100% similarity for subtilosin A, a known antibacterial protease (Fig. S5, S6). The *sboA-alb* operon, associated with subtilosin A production, was predicted in the 2AT10 genome. Sequence alignment of the *sboA* gene with *sboA* genes from *B. subtilis* strains revealed the same SboA1 mutation previously described by Huang et al. (2009) involving a substitution of threonine with isoleucine at position 6. In addition, regulatory proteins encoded by *albG* and *albD*, which are involved in subtilosin A biosynthesis, exhibited multiple amino acid substitutions (15 and 6 replacements, respectively) when compared with homologous sequences available in UniProt Table S6.

**Table 5:** Gene clusters and respective information (type, size, position in genome, most similar known cluster, and similarity) of secondary metabolites using antiSMASH analysis

Type	Size (nt)	Position	Most similar known cluster		Similarity
Terpene, T3PKS, RiPP-like	1098	188.815 – 189.912	1-carbapen-2-em-3-carboxylic acid	Other	16%
Sactipeptide	132 1347	404.394 – 404.525 404.659 – 406.005	Subtilosin A	RiPP: Thiopeptide	100%
Other RIPP-like terpene	1419	439.304 – 440.722	Bacilysin	Other	100%
NRPS, T1PKS	4497	82.229 – 86.725	Zwittermicin	NRP+Polyketide	18%
NRP-metallophore, NRPS	7137 1197 786	101.705 – 108.841 111.475 – 112.671 112.697 – 113.482	Bacillibactin	NRP	100%
NRPS	3828	1.379 – 5.206	Surfactin	NRP: Lipopeptide	43%
NRPS	7710	1 – 7.710	Surfactin	NRP: Lipopeptide	43%
TransAT-PKS, NRPS, T3PKS, PKS-like	906 975 2304 1248 1263	8.143 – 9.048 9.548 – 10.522 10.519 – 12.822 13.110 – 14.357 14.358 – 15.620	Bacillaene	Polyketide + NRP	100%
NRPS, betalactone	900 1650	178.266 – 179.165 180.760 – 182.409	Fengycin	NRP	73%
NRPS	7680 3855	1.577 – 9.256 9.281 – 13.135	Plipastatin	NRP	23%
NRPS	3513 5769	1 – 3.513 3.539 – 9.307	Fengycin	NRP	20%
NRPS	7698	1.355 – 9.052	Surfactin	NRP: Lipopeptide	8%

Some low similarities observed in Table 5 could be related to the low number of genes in the bacterial gene clusters of the specific compound. Clusters classified as “Other” or “RiPP-like”, such as the one associated with 1-carbapen-2-em-3-carboxylic acid (with 16% similarity), are not commonly associated with Gram-positive strains but were also identified. Furthermore, using the EggNOG mapper functional annotation tool (Supplementary Dataset S1), a gene related to lactococcin production (named BsLac in this article) was identified with highly significant e-value of 1.67e-66 and a bit score of 210, indicating strong sequence similarity to functionally characterized homologs. Sequence homology analysis (Fig. S7a) confirmed 100% coverage and 40% amino acid identity with a Lactococcin 972 family bacteriocin from *Lactococcus* (NCBI ID: WP_017863933.1).

### Computational bioactivity prediction

**Fig. 5:**
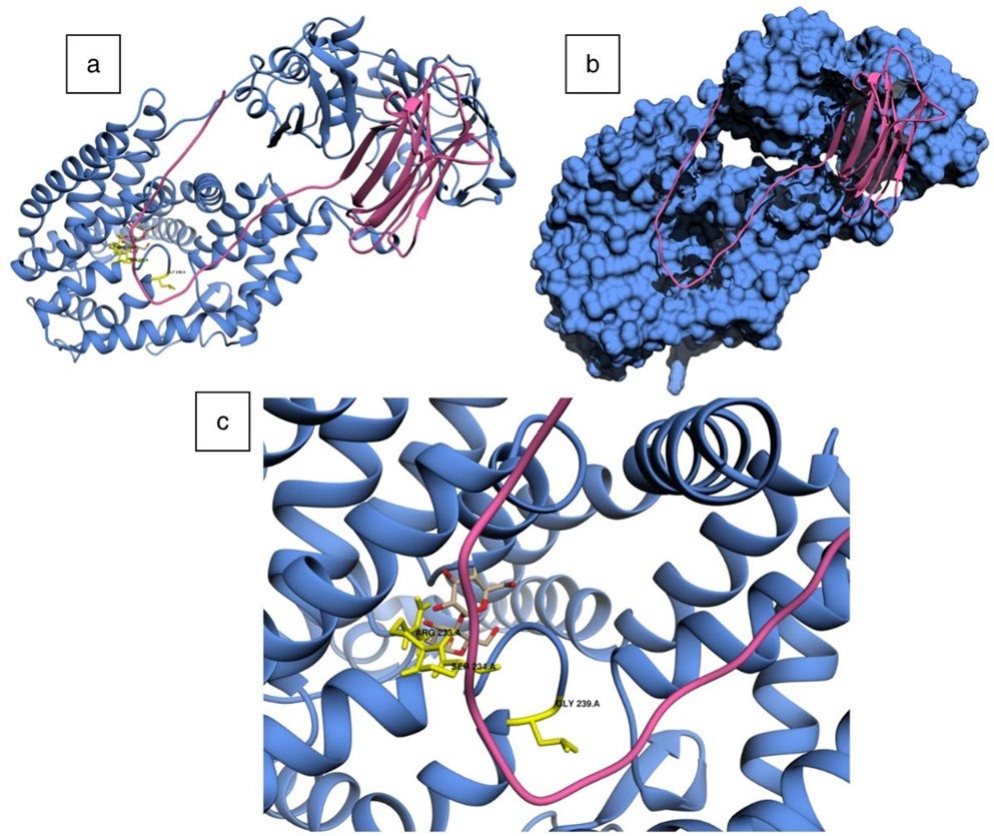
Molecular docking results of the complex between GlcB from *S. aureus* and the lactococcin-like BsLac from *Bacillus stercoris* AT210. **(a)** Overall docking positioning of BsLac (pink) inside the GlcB (blue) TM7 region. The helical TM7 amino acids Arg233, Ser234, and Gly239 are highlighted in yellow. **(b)** Overall docking positioning of BsLac (pink), showing the structure of GlcB (blue) in surface mode. This view shows that the entire BsLac terminal coil is inside the TM pocket, particularly within the TM7 helices. **(c)** Detailed view of the BsLac (pink) coil inside the GlcB (blue) TM7 pocket, with the highlighted amino acids Arg233, Ser234, and Gly239 in yellow. A glucose molecule is highlighted in brown inside the active site of GlcB

Molecular docking results for the protein-protein complex GlcB*-*BsLac returned an energy score of -114 Kcal/mol, with the BsLac terminal coil region (Met1 to Asp27) inside the TM7 portion of the GlcB protein (Fig. 5a and b**)**. A detailed docking visualization (Fig. 5c) indicated that BsLac possibly accesses the GlcB transporter through its outer membrane face, introducing the C-terminal coil region close to the TM7 helices of GlcB. Hence, amino acids such as Arg233, Ser234, and Gly239 were found facing the BsLac C-terminal coil and surrounding the glucose transporter’s active site. Interestingly, this docking position is similar to the protein used as a template for defining the GlcB docking region (5IWS), as observed in the structural alignment between the GlcB*-*BsLac complex and the template of Fig. S7 a and b.

The molecular dynamics of stability for the GlcB*-*BsLac complex indicated that during the 500 ns simulation, BsLac remained inside the TM region of the GlcB protein, close to the region proposed for the docking experiments. In addition, the RMSD graph comparing the Apo and Holo (Fig. S8A) forms of the transporter GlcB indicates that the conformational changes, with an increase in the Holo RMSD backbone during the first 250 ns, could suggest a mechanism similar to a closed state of this protein induced by the presence of the terminal coil of BsLac inside the TM region. Furthermore, the analysis of the RMSF (Fig. S8B) showed that important amino acids related to the TM7 and active site pockets of the transporter exhibited lower fluctuations in the complex compared to the transporter in its Apo form, indicating that the presence of BsLac could block this region and interfere with glucose passage in this way. In addition, hydrogen bonds formed between both proteins in the complex remained constant throughout the entire MD simulation, ranging from 2 to 20 per frame of 100 picoseconds (Fig. S8C, S8D).

GROMACS energies from Lennard-Jones and Coulomb parameters showed that the complex form (Holo) was favorable in both graphs (Fig. S8E, S8F), showing negative values ranging from 6.1 × 10⁵ kJ/mol to 6.5 × 10⁵ kJ/mol for the first one, and from 4.22 × 10⁶ kJ/mol to 4.18 × 10⁶ kJ/mol for the second. These numbers could indicate that the GlcB*-*BsLac complex is energetically favorable compared to the Apo form. Furthermore, the system’s total energy is also negative, suggesting that interaction in the complex could be naturally possible.

## Discussion

Limited published research exists on *B. stercoris*, likely due to its recent reclassification. This classification highlights its close evolutionary relationship with *B. subtilis*, suggesting that these two species could be closely related with respect to their functional genome annotation and potential microbial activities (Chouaia and Dittmer 2024, Dunlap et al. 2020).

Between 2014 (the year of the first deposit) and 2024, 17 genomes for *B. stercoris* or *B. subtilis subsp. stercoris* were submitted to GenBank. Among these, only four genomes have corresponding data published in scientific articles. The first reports on these species appeared in PubMed in 2019, with 21 articles available by 2024. Of these publications, eight manuscripts specifically examine antimicrobial potential. Out of these publications, just two correlate genomic data with *in vitro* analyses, while the remainder focus on biotechnological applications, particularly enzymatic activity and stress tolerance.

The isolate 2AT10, identified as *B. stercoris* in this study, demonstrated strong *in vitro* antimicrobial activity against various ATCC reference strains and drug-resistant clinical bacterial isolates (Gram-positive and Gram-negative), including multiple drug-resistant *S. aureus* isolates. Studies show that due to the presence of the outer membrane, Gram-negative microorganisms are less susceptible to antimicrobial agents due to reduced permeability, which may explain the reduced spectrum of inhibition (Lehman and Grabowicz 2019, MacNair and Brown 2020). The *in vitro* antimicrobial activity observed in this study, may be associated with the combined effect of multiple metabolites predicted in the genome; however, their individual contributions remain to be experimentally determined (Chouaia and Dittmer 2024, Wang et al. 2021, Ye et al. 2023, Ku et al. 2024).

The antibacterial activity of the supernatants analyzed in this study was more pronounced against Gram-positive bacteria which could be associated with the presence of the mutated sboA gene involved in subtilosin A biosynthesis; however, this relationship remains speculative in the absence of compound-level validation. The study of Huang et al. (2009) describing the first occurrence of this mutation in the *sboA* gene in *B. subtilis* reported hemolytic activity associated with increased activity against Gram-positive bacteria, similar to our *in vitro* and i*n silico* results.

The terpene cluster (associated with carbapenem antibiotic) is commonly present in Gram-negative organisms such as *Serratia* sp. and *Erwinia* sp (Bycroft et al. 1988). A terpene cluster (16% identity) in the 2AT10 genome might represent novel biosynthetic pathways that are not yet characterized (McGowan et al. 1997).

The reduction in antimicrobial activity following protease treatment suggests that proteinaceous components contribute to the observed bioactivity in both supernatants S and SP. This observation is consistent with the FTIR data, which indicates the presence of functional groups commonly associated with protein-containing mixtures (Villa-Rodriguez et al. 2021, Mujaddidi et al. 2021, Yadav et al. 2023). Fugaban et al. (2024) and Amiri et al. (2022), working with *B. stercoris* and *Bifidobacterium lactis*, have also reported bacterial supernatants that are enzymatic and thermic, as well as pH treatment sensitive. It is noteworthy to mention here that most secondary metabolites gene clusters found in the *B. stercoris* 2AT10 genome, such as bacillaene and fengycin, are reported to be temperature sensitive (Li et al. 2021, Gimenez et al. 2021), while subtilosin A and bacilysin are reported to be thermal, enzymatic, and pH stable (Alajlani 2022, Abdulmalek and Yazgan-Karataş 2023). One of our hypotheses to explain the differences between supernatants S and SP is related to the distinct methods used for metabolite recovery. Supernatant S likely contains compounds actively secreted during bacterial growth, such as lipopeptides and other diffusible molecules, whereas SP is expected to be enriched in membrane-associated compounds released during the washing step.

The supernatants S and SP analyzed showed no irritative effect in the HET-CAM test. Other studies, such as that by Wang et al. (2024), also using HET-CAM, demonstrated that products of microbial metabolism are non-irritative. Many agents with antibiotic properties, such as tetracycline, exert a slightly irritating effect on the chicken chorioallantoic membrane. At the same time, ampicillin has a stronger irritative effect, according to a study by Hut et al. (2021). The HET-CAM assay has been proposed as a reliable alternative due to its highly vascularized and functional membrane, making it comparable to the rabbit eye test. HET-CAM is also preferred for being faster, more cost-effective, and cruelty-free. Additionally, quantitative methods like the MTT assay assess toxicity levels. Still, studies suggest that the advantages of HET-CAM, such as lower cost and reduced toxic waste, make it a viable option, with no significant difference in accuracy compared to the MTT assay (Uner et al. 2023).

Genome mining using antiSMASH and BAGEL enabled the identification of several biosynthetic gene clusters, showing high similarity to previously characterized clusters. In contrast, the BsLac protein was not detected within a canonical biosynthetic gene cluster by these tools. Its identification was instead based on functional annotation using EggNOG-mapper, which revealed significant sequence similarity to members of the Lactococcin 972 family. This type of annotation relies on homology-based inference rather than conserved cluster architecture. The absence of BsLac from antiSMASH and BAGEL outputs may reflect the limited representation of certain less characterized bacteriocin classes or atypical genetic organizations in current databases (Medema et al. 2011, Cantalapiedra et al. 2021). Lactococcins are less characterized within the genus *Bacillus*, thus forming the basis of the central hypothesis of this study as a potentially novel protein in *B. stercoris*. Structural alignment between the lactococcin 972 produced by *Lactococcus* and BsLac revealed 40% amino acid identity, suggesting functional conservation between those proteins.

Phosphotransferase system (PTS) transporters can mediate the uptake and phosphorylation of various sugars, such as glucose and mannose (Ge et al. 2024). Integral membrane components of PTS transporters (represented in this study by GlcB of *S. aureus*), which are involved in sugar translocation, normally act as receptors for class II bacteriocins (Jeckelmann and Erni 2020). In addition, Lactococcin 972 is a class IId bacteriocin, which has been described as causing lactococcal membrane damage by disrupting PTS after direct interaction (Hernández-González et al. 2021, Holo et al. 1991, Li et al. 2023). PTS transporters are common in Gram-positive bacteria such as *S. aureus*. In this study, GlcB was chosen as the only PTS-associated transporter protein structure for *S. aureus* (bacteria focused on the *in vitro* results) available in UniProt. Our *in silico* molecular docking and dynamics results predicted an interaction between BsLac and GlcB in *S. aureus*, which could be associated with higher antimicrobial activity observed in our study; however, no direct evidence currently links this predicted interaction to the antimicrobial activity observed *in vitro*. One of our hypotheses is that BsLac binding to GlcB could lead to the leakage of intracellular and transmembrane components, which are essential for membrane integrity, by increasing permeability and inducing pore formation, as suggested by Zhu et al. (2022) and by Li et al. (2023). The absence of the PTS system in Gram-negative bacteria may have influenced the less significant results of *in vitro* antimicrobial activity.

Different authors have reported that, usually, the C-terminal loop regions of the class II lactococcins are responsible for blocking the mechanism of PTS transport (Hernández-González et al. 2021, Li et al. 2023, Tymoszewska and Aleksandrzak-Piekarczyk 2024). As we reported in Fig. S7, the C-terminal region of this type of lactococcin is commonly responsible for the penetration into the protein receptor by the face turned to the outer membrane, where it reaches a transmembrane region (Li et al. 2023, Tymoszewska and Aleksandrzak-Piekarczyk 2024). Furthermore, it is possible to suggest that like other studies with class IId bacteriocins, the mechanism of action of BsLac faces of the *S. aureus* glucose PTS transporter would block the open-close state mechanism of this kind of protein, as described for the 5IWS template, which is crucial for glucose intake. As described in our study, by entering the C-terminal region close to the TM5 and TM7 regions, BsLac could turn this receptor permanently closed to the passage of the sugar inside the bacterial cytoplasm (McCoy et al. 2016).

The activity of lactococcins against *S. aureus* has not been explicitly reported (*in vitro* or *in silico*), although several studies have suggested their potential role in co-cultures with lactic acid bacteria targeting this pathogen (Hasan et al. 2025, Yaacob et al. 2022, Darbandi et al. 2022). Lactococcins A, B and M, for example, are effective against *Lactococcus*, while lactococcin BZ from *L. lactis* spp. inhibited Gram-negative bacteria growth such as *Listeria monocytogenes* and *Escherichia coli* (Darbandi et al. 2022, Yildirim et al. 2016, Öncül and Yıldırım 2019). Lactococcin 972-like bacteriocin, named as Lcn972, isolated and purified from *Bacillus velezensis* showed activity against various *Bacillus* spp., *Streptomyces* spp. related with soil pathogenic bacteria (Zhao et al. 2022). Our *in silico* analyses provide first preliminary insights into a possible interaction between the predicted lactococcin-like protein (BsLac) and GlcB in *S. aureus*, suggesting a potential mechanism of action that requires experimental validation.

Lactococcin and subtilosin A are known to disrupt the cell membrane (Tymoszewska and Aleksandrzak-Piekarczyk 2024); therefore, their combined effect may lead to enhanced membrane disruption and increased bacterial cell death. Based on this, we hypothesize a potential synergistic effect between BsLac and subtilosin A that may contribute to the enhanced activity observed against Gram-positive bacteria; however, this remains speculative and requires experimental validation.

## Conclusions

The Gram-positive, spore-forming isolate *Bacillus stercoris* 2AT10, obtained from coastal sand, demonstrated relevant antimicrobial activity, particularly against Gram-positive bacteria, including methicillin-resistant MRSA. The observed bioactivity is associated with crude supernatant fractions and likely reflects the combined effect of multiple metabolites produced during bacterial growth. Genome analysis revealed the presence of several biosynthetic gene clusters related to known antimicrobial compounds, supporting the potential of this strain as a source of bioactive molecules. In addition, a lactococcin-like sequence was identified through functional annotation; however, its production, activity, and contribution to the observed antimicrobial effects remain to be experimentally validated. *In silico* analyses suggested a possible interaction between the predicted lactococcin-like protein (BsLac) and a membrane-associated transporter in *S. aureus*. Nevertheless, these findings are hypothesis-generating and should be interpreted with caution, as no direct experimental evidence currently supports this interaction or its role in antimicrobial activity. Given the recent reclassification and limited characterization of *B. stercoris*, further studies are required to isolate and characterize the active compounds, validate their mechanisms of action, and determine their potential applicability. Future work should focus on compound purification, quantitative activity assessment, and experimental validation of the predicted molecular interactions.

## Supplementary Information

Below is the link to the electronic supplementary material.

Supplementary Material 1

Supplementary Material 2

## Data Availability

The datasets generated and/or analyzed during the current study are present in the current manuscript and supplementary information provided. Any other extra information regarding the data sets generated and/or analyzed is available from the corresponding author on reasonable request. Sequence data that supports the findings of this study is deposited in the GenBank with Gen Bank ID GCA\_041765565.1, RefSeq ID GCF\_041765565.1.
