# Supplementary material for: Genome-guided antimicrobial potential of Bacillus stercoris from coastal sand with activity against multidrug-resistant bacteria, including MRSA
Source: Int Microbiol. 2026 May 1;29(5):713–28. doi: 10.1007/s10123-026-00836-x (PMC13260041; doi:10.1007/s10123-026-00836-x)
Supplement: Supplementary file 2 — Supplementary Material 2 [file 10123_2026_836_MOESM2_ESM.docx]

**SUPPLEMENTARY MATERIAL (Figures)**


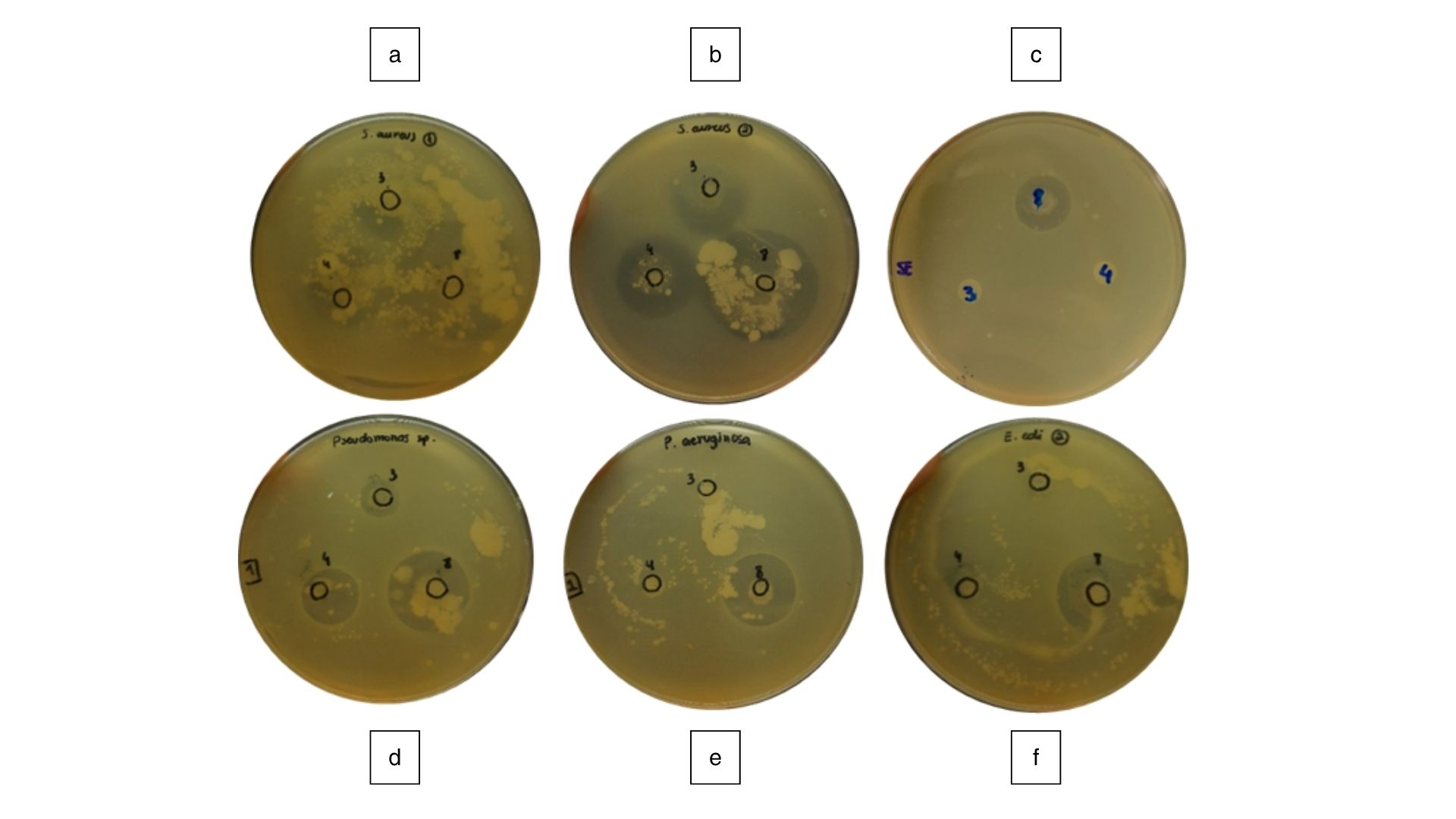


**Fig. S1** Results of the agar overlay test of isolate 2AT10 (marked as 8) from the beach sand, against Gram-positive and negative pathogenic indicators: **A)** *S. aureus* ATCC 8095; **B)** *S. aureus* ATCC 25923; **C)** *S. epidermidis* ATCC 12228; **D)** *P.* *aeruginosa* ATCC 27853; **E)** *P. aeruginosa* ATCC 27583; **F)** *E. coli* ATCC 25922.


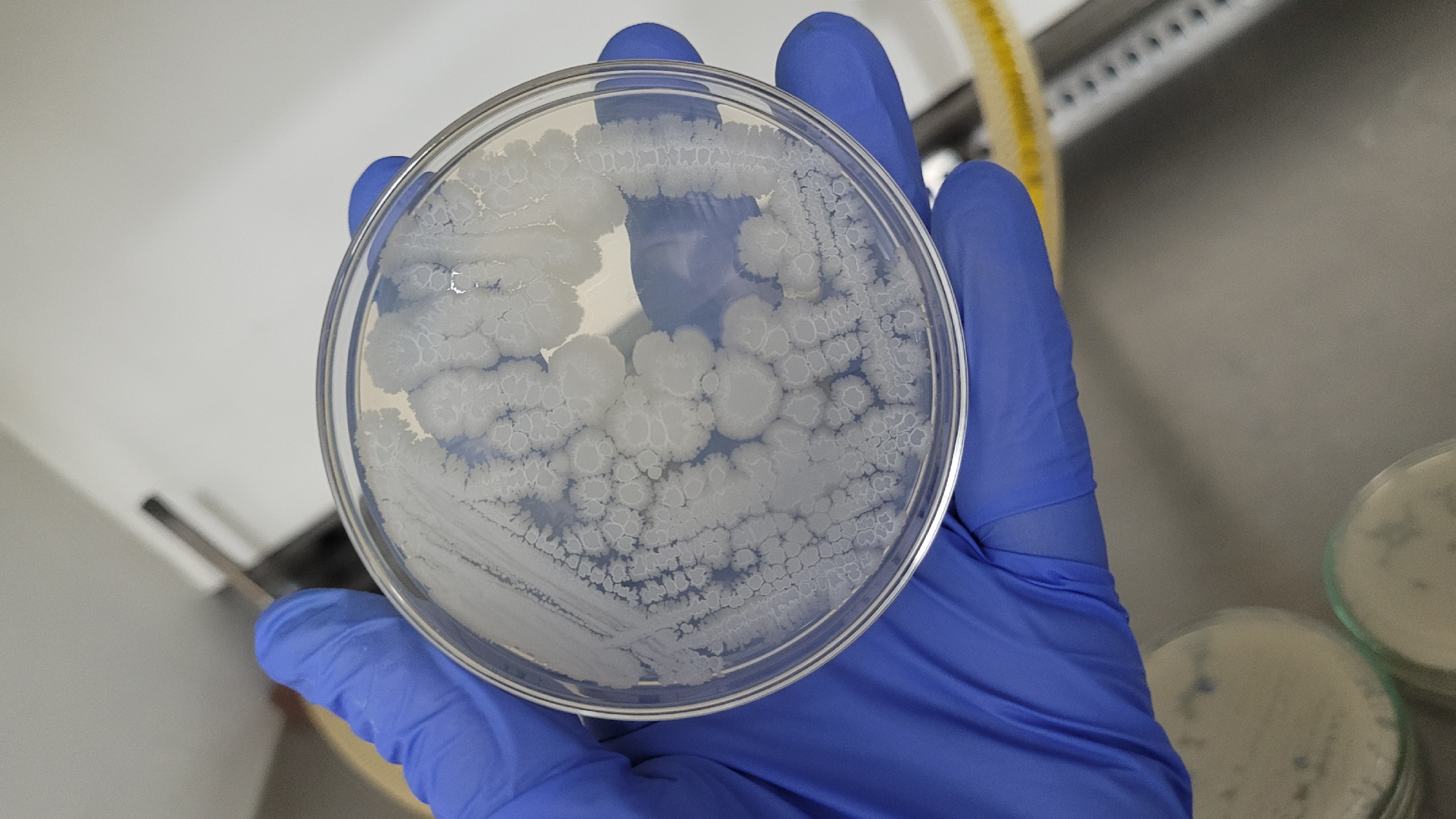


**Fig. S2** Bacterial isolate 2AT10 growing on nutrient agar.


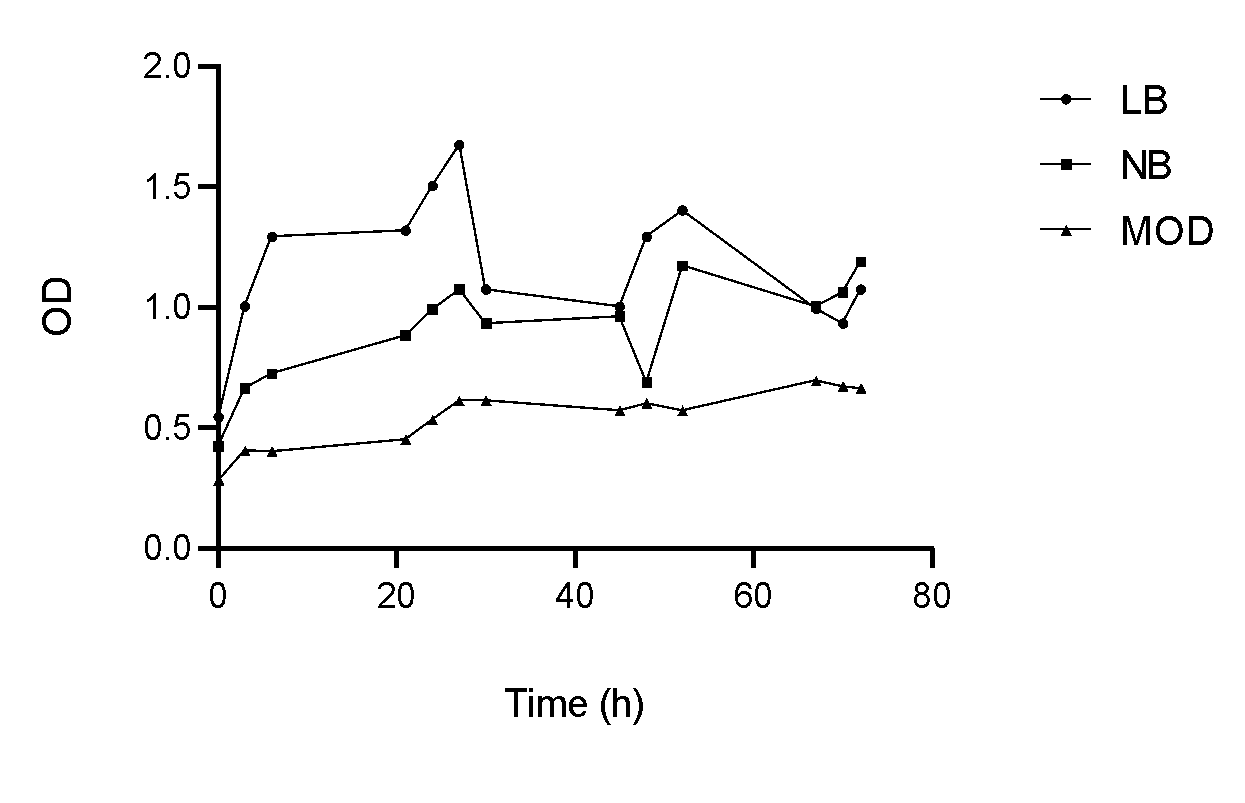


**Fig. S3** 2AT10 growth curve with standard deviation in Luria-Bertani Broth (LB), Nutrient Broth (NB) and Modified Medium (MOD). Bacterial growth was monitored by measuring the optical density (OD) at 600 nm.


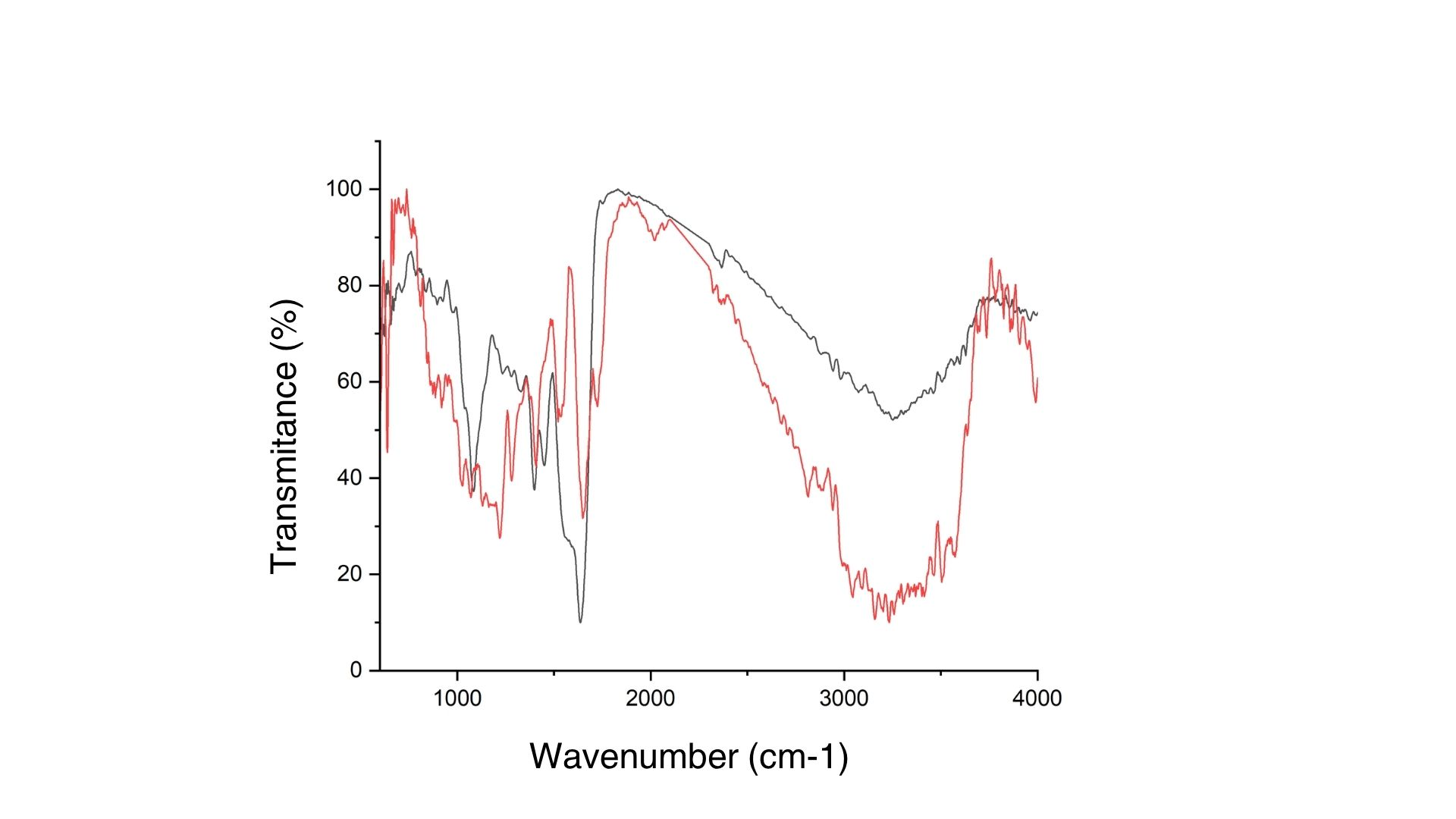


**Fig. S4** Fourier-Transform Infrared Spectroscopy profiles of supernatants: S (black line) and SP (red line).


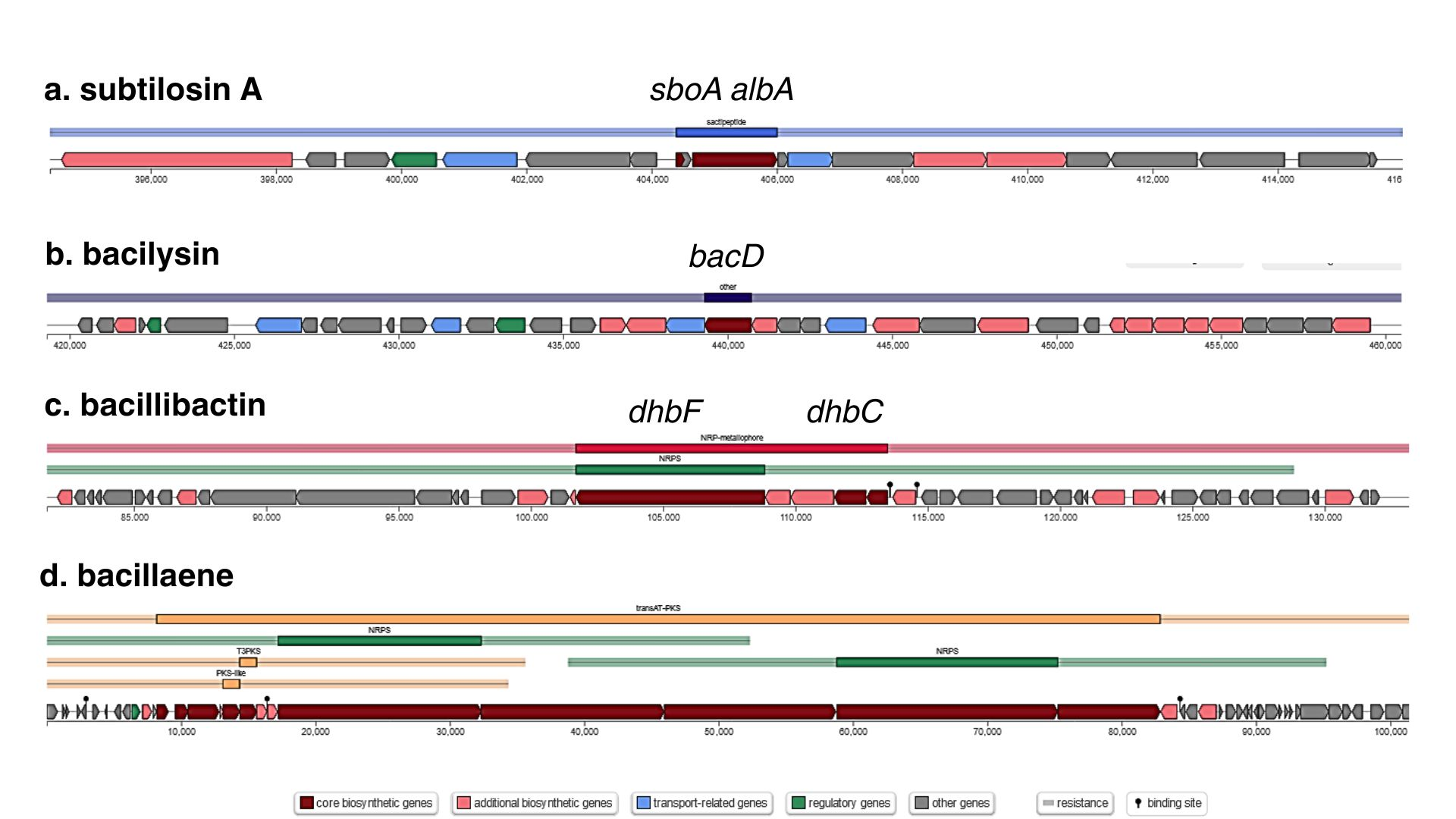


**Fig. S5** AntiSMASH 7.1.0 analysis of **(a)** subtilosin A, **(b)** bacilysin, **(c)** bacillibactin, and **(d)** bacillaene gene clusters (>80% similarity) of *B. stercoris* 2AT10.


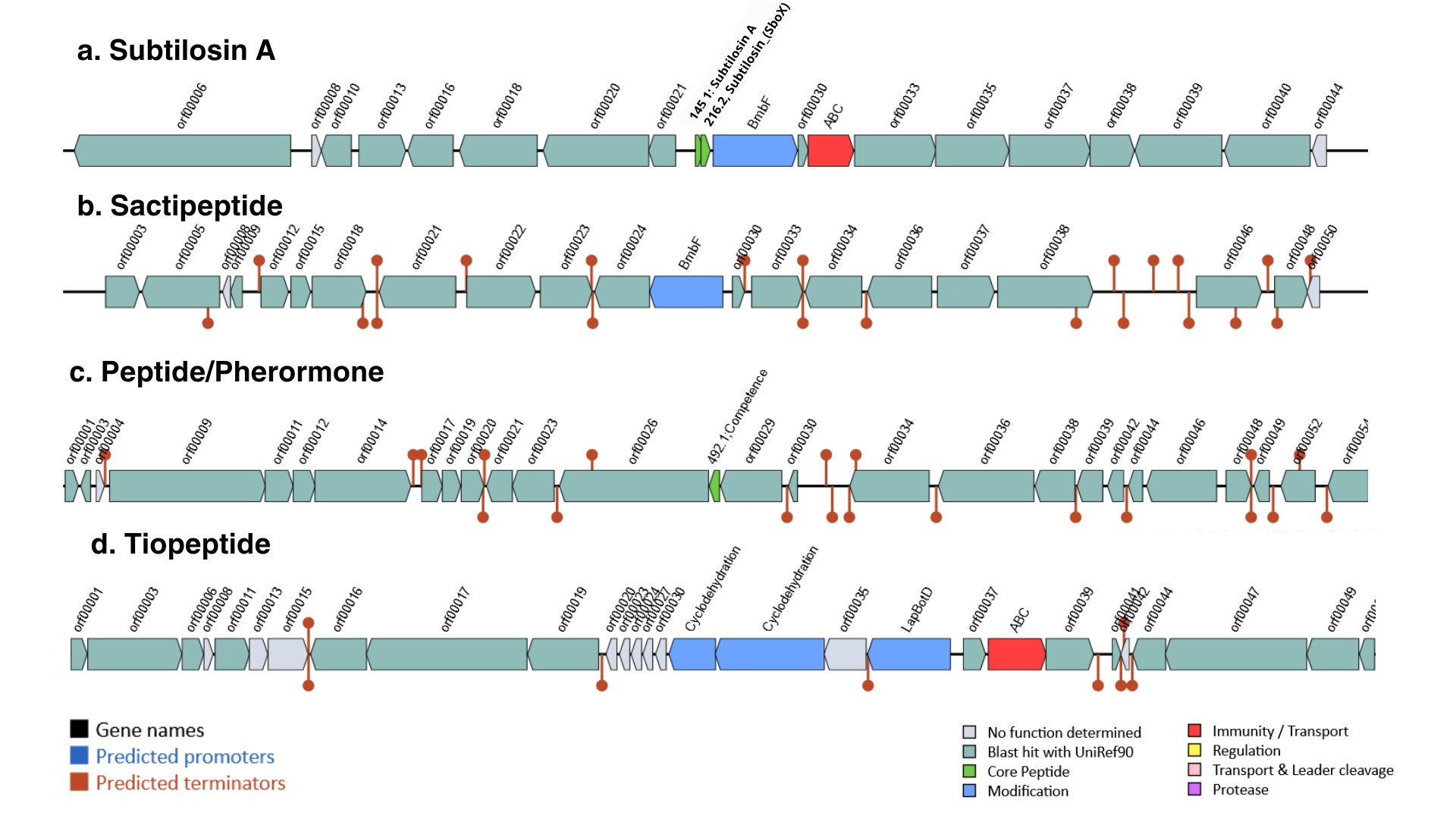


**Fig. S6** Genes clusters of *B. stercoris* 2AT10 from BAGEL analysis associated with secondary metabolites production.

**Fig. S7 A)** Alignment between Lactococcin from *Lactococcus* and BsLac with 100% coverage and 40 % homology through T-coffee tool. **B)** Structural alignment between the GlcB (blue) - BsLac (pink) complex and the PDB template 5IWS (grey). All the helices are aligned between both transporters, while BsLac appears inside the TM7 region
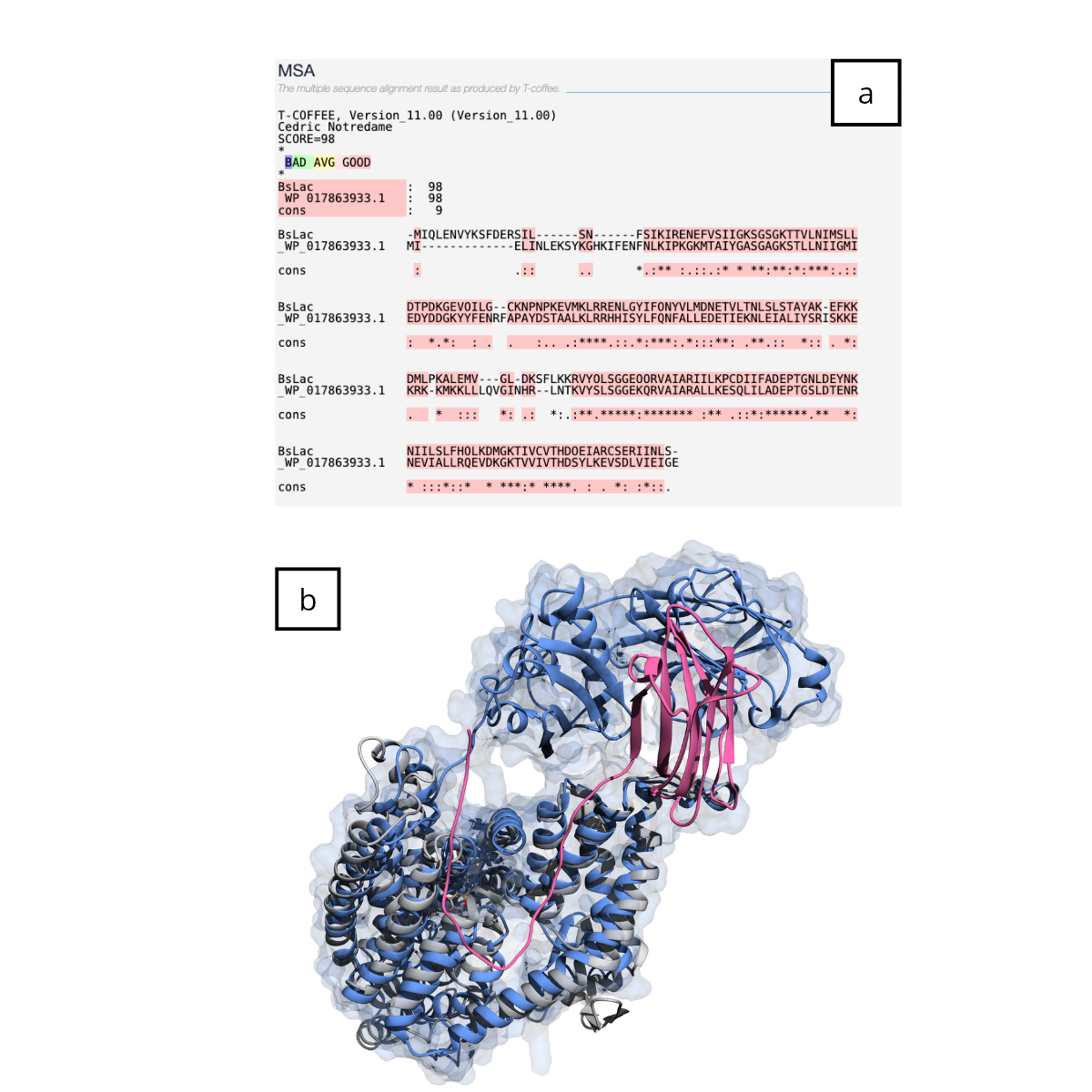


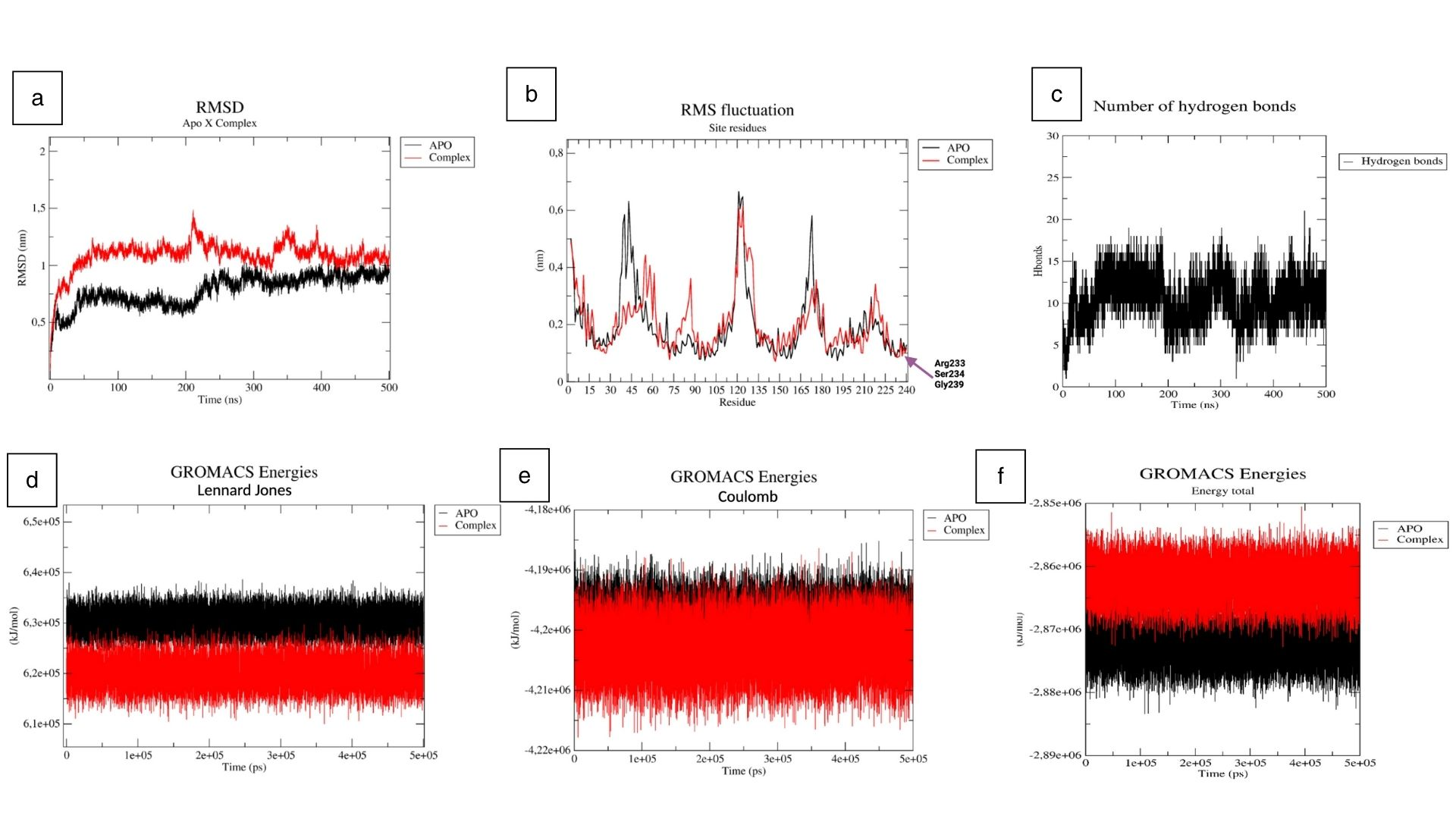


**Fig. S8** 500 ns GROMACS molecular dynamics simulation for both GlcB in its Apo form (black lines) and complex (red lines) with BsLac (Holo). **A)** Root Mean Square Deviation (RMSD) graph comparing the protein backbones. **B)** Root Mean Square Fluctuation (RMSF) graph analyzing the deviation of TM7 amino acids close to the GlcB active site. **C)** Number of hydrogen bonds formed during the MD simulation per 100-picosecond frames. **D)** Lennard-Jones potential energy graph describing the variation of potential energy between pairs of atoms in the system. **E)** Coulomb potential energy graph showing the variation of electrostatic potential energy between pairs of charged particles in the system. **F)** Total energy of the GlcB system in Apo and Holo forms.

**SUPPLEMENTARY MATERIAL (Tables)**

**Table S1** Accession number of clinical isolates drug-resistant used for antimicrobial activity tests.

| Species | Strains | Gram | Phenotype | MDR bacteria | Accession number |
| --- | --- | --- | --- | --- | --- |
| *Staphylococcus aureus* | IOA14 | Positive | MSSA | No | NA |
|  | UAMS1 | Positive |  |  | JTJK00000000 |
|  | Newman | Positive |  |  | AP009351 |
|  | S20 | Positive |  |  | LVZU00000000 |
|  | Thom | Positive |  |  | LVZX00000000 |
|  | P19 | Positive |  |  | LAWS00000000 |
|  | C57 | Positive |  |  | JYEM00000000 |
|  | P8 | Positive |  |  | JYEV00000000 |
|  | USA300 LAC | Positive | MRSA | Yes | CP000255.1 |
|  | P333 | Positive |  |  | LSGO00000000 |
|  | S43 | Positive |  |  | LJBD00000000 |
|  | C26 | Positive |  |  | JYEJ00000000 |
|  | CA1 | Positive |  |  | ST20160825 |
|  | LA1 | Positive |  |  | NA |
|  | LA2 | Positive |  |  | NA |
|  | Van 035 | Positive | MSSA/VRSA | Yes | NA |
|  | van 023 | Positive |  |  | LVZP00000000 |
|  | Mu50 | Positive | MRSA/GISA | Yes | GCA_000009665 |
| *Staphylococcus haemolyticus* | STHA1 | Positive | Meti-R, Vanco-S | Yes | PRJEB72027 |
|  | STHA2 | Positive | Meti-R, Vanco-R | Yes | PRJEB72027 |
| *Enterococcus faecium* | Aus 004 | Positive | VRE | Yes | CP003351 |
| *Klebsiella pneumoniae* | KLPN1 | Negative | ESBL | Yes | NA |
| *Enterobacter cloacae* complex | ATCC 13047 | Negative |  |  | CP001918 |
|  | ECC1 | Negative | ESBL-CPE | Yes | NA |
| *Citrobacter freundii* | CIFR1 | Negative | ESBL-CPE | Yes | NA |

NA: Not applicable; MSSA: Methicillin-Sensitive *Staphylococcus aureus*; MRSA: Methicillin-Resistant *Staphylococcus aureus*; VRSA: Vancomycin-Resistant *Staphylococcus aureus*; GISA: Glycopeptide-Intermediate *Staphylococcus aureus*; Meti-R: Methicillin-Resistant; Vanco-S: Vancomycin-Sensitive; VRE: Vancomycin-Resistant *Enterococcus*; ESBL: Extended-spectrum beta-lactamases; ESBL-CPE: Extended-spectrum beta-lactamases and Carbapenemase-producing *Enterobacteriaceae*.

**Table S2** Public Genomes of *Bacillus stercoris* and six *Bacillus* sp. outgroups from NCBI used in genomic comparation

| *Bacillus stercoris* | | |
| --- | --- | --- |
| Strain | **Assembly Accession** | **Organism Name** |
| 4.5 | GCF_030315825.1 | *Bacillus stercoris* |
| B-3280 | GCF_035813135.1 | *Bacillus stercoris* |
| B-3281 | GCF_035813075.1 | *Bacillus stercoris* |
| BD-618 | GCF_035822295.1 | *Bacillus stercoris* |
| BHUJPV-SS7 | GCF_022814905.1 | *Bacillus stercoris* |
| BS21 | GCF_029958885.1 | *Bacillus stercoris* |
| BST19 | GCF_037101185.1 | *Bacillus stercoris* |
| Bam 2 | GCF_034427695.1 | *Bacillus stercoris* |
| D7XPN1 | GCF_000738015.1 | *Bacillus stercoris* |
| DHFl4 | GCF_020551885.1 | *Bacillus stercoris* |
| Mal05 | GCF_029772985.1 | *Bacillus stercoris* |
| PSM7 | GCF_023703335.1 | *Bacillus stercoris* |
| SEN 14.5 | GCF_030553605.1 | *Bacillus stercoris* |
| SMPL712 | GCF_030219925.1 | *Bacillus stercoris* |
| ZBMF30 | GCF_030846295.1 | *Bacillus stercoris* |
| ZBOE3 | GCF_039770125.1 | *Bacillus stercoris* |
| Other *Bacillus* species (outgroups) | | |
| DSM7 | GCF_000196735.1 | *Bacillus amyloliquefaciens* |
| KCTC 13429 | GCF_003148415.1 | *Bacillus inaquosorum* |
| TU-B-10 | GCF_000227465.1 | *Bacillus spizizenii* |
| ATCC 6051 | GCF_000186085.1 | *Bacillus subtilis* |
| NCTC 13306 | GCF_900445435.1 | *Bacillus tequilensis* |
| DSM 11031 | GCF_004116955.1 | *Bacillus vallismortis* |

**Table S3** Antibiogram of the isolate 2AT10 according to CLSI (2023).

| **Antibiotic (μg)** | **Inhibition (mm)** | **Result** |
| --- | --- | --- |
| AMO 10 | 35 | S |
| GEN 10 | 27 | S |
| CLO 30 | 30 | S |
| CFX 30 | 33 | S |
| CIP 5 | 36 | S |
| NEO 30 | 26 | S |
| OXA 1 | 12 | S |
| VAN 30 | 20 | S |
| NOR 10 | 33 | S |

**Table S4** Inhibition zone diameters of supernatants S and SP against *S. aureus* ATCC 25923 and *Pseudomonas* *aeruginosa* ATCC 27853 thermal, enzymatic and pH stability assays.

| **Stability Test** | ***S. aureus* (ATCC 25923)** | | ***P. aeruginosa* (ATCC 27853)** | |
| --- | --- | --- | --- | --- |
|  | **S** | **SP** | **S** | **SP** |
| **Thermal** | | | | |
| 40°C | 6.16 ± 0.15* | 6.11 ± 0.1 | 4.2 ± 0.2* | 4.21 ± 0.07 |
| 50°C | 4.15 ± 0.13* | 4.21 ± 0.12 | 3.2 ± 0.23* | 3.23 ± 0.23 |
| 60°C | 3.08 ± 0.1* | 3.18 ± 0.12* | 2.2 ± 0.1* | 2.16 ± 0.1* |
| 70°C | 1.6 ± 0.1* | 1.65 ± 0.13* | 1.01 ± 0.07* | 1.48 ± 0.12* |
| 80°C | - | - | - | - |
| 90°C | - | - | - | - |
| 100°C | - | - | - | - |
| **Enzymatic** | | | | |
| Proteinase K (1h treatment) | 5.13 ± 0.23 | 4.58 ± 0.29 | 3.93 ± 0.11 | 3.41 ± 0.07 |
| Proteinase K (2h treatment) | 3.4 ± 0.2 | 4.08 ± 0.1 | 2 ± 0.1 | 2.53 ± 0.23 |
| **pH** | | | | |
| pH 2 | 5.2 ± 0.21* | 10.7 ± 0.2 | - | 6.01 ± 0.12 |
| pH 5 | - | 4.06 ± 0.11 | - | 2.8 ± 0.15 |
| pH 7 | 4.03 ± 0.15* | - | 3.18 ± 0.17* | - |
| pH 10 | - | 2.15 ± 0.21 | - | 1.18 ± 0.16 |
| **Control (**supernatants without treatment**)** | 10.11 ± 0.1 | 10.7 ± 0.2 | 5.7 ± 0.3 | 6.01 ± 0.12 |

* Clear inhibition zone not observed

- no activity

**Table S5** Minimum information about the genome sequence (MIGS) of *Bacillus stercoris* 2AT10

| Item | Description |
| --- | --- |
| Project Name | *Bacillus stercoris* 2AT10 Genome sequencing and assembly |
| Organism | Bacteria; Firmicutes; Bacilli; Bacillales;  Bacillaceae; *Bacillus; B. stercoris* 2AT10 |
| Sample type | Chromosomal DNA, plasmid DNA |
| Collection date | 2021-04-10 |
| Geographic location (country and/or sea) | Brazil, Aracaju |
| Geographic location (latitude) | 10°58'43.6"S |
| Geographic location (longitude) | 37°02'11.7"W |
| Environment (biome) | Aquatic |
| Environment (feature) | Beach Sands |
| Environment (material) | Soil |
| Geographic location | -10.978362678194104, -37.03544618623954 |
| Sequencing Platforms | Illumina HiSeq 2500 |
| Assembly method | Unicycler v. 0.5.0 |
| Investigation type | Bacterial Genome sequencing and annotation |
| Finishing Quality | Contigs (40) |
| Fold Coverage | 606x |
| Gen Bank ID | ASM4176556v1 |

**Table S6** Amino acid substitutions in the AlbG and AlbD protein sequences showing new amino acids introduced at specific positions and the original amino acids observed at those positions, based on UniProt sequences.

| AlbD | | |
| --- | --- | --- |
| Replacement | **Position** | **Original** |
| Serine | 3 | Asparagine/Isoleucine |
| Serine | 6 | Proline/Methionine/Lysine |
| Asparagine | 48 | Aspartic acid/Alanine/Threonine |
| Alanine | 317 | Methionine/Glutamic acid/Serine |
| Threonine | 405 | Alanine/Lysine/Leucine |
| Leucine | 409 | Proline/Isoleucine/Tyrosine |
| AlbG | | |
| Replacement | **Position** | **Original** |
| Arginine | 2 | Serine/Lysine |
| Tyrosine | 30 | Alanine |
| Serine | 40 | Asparagine/Tryptophan |
| Valine | 127 | Isoleucine/Phenylalanine |
| Tyrosine | 144 | Histidine |
| Leucine | 173 | Phenylalanine |
| Valine | 209 | Leucine |
| Serine | 219 | Phenylalanine |
| Histidine | 222 | Arginine |
| Isoleucine | 223 | Phenylalanine |
| Asparagine | 225 | Asparagine/Tyrosine |
| Arginine | 227 | Glycine/serine |
| Proline | 228 | Phenylalanine/Threonine |
| Phenylalanine | 231 | Leucine |
| Glutamic Acid | 232 | Aspartic acid |
